# Reconstruction of large-scale regulatory networks based on perturbation graphs and transitive reduction: improved methods and their evaluation

**DOI:** 10.1186/1752-0509-7-73

**Published:** 2013-08-08

**Authors:** Andrea Pinna, Sandra Heise, Robert J Flassig, Alberto de la Fuente, Steffen Klamt

**Affiliations:** 1 Center for Advanced Studies, Research and Development (CRS4) Bioinformatica, Pula, Italy; 2 Max Planck Institute for Dynamics of Complex Technical Systems, Magdeburg, Germany; 3 Leibniz Institute for Farm Animal Biology, Dummerstorf, Germany

**Keywords:** Gene network inference, Reverse engineering, Perturbation experiments, Causal networks, Graph theory, Interaction graphs, Transitive reduction, Transcriptional regulation, Saccharomyces cerevisiae, Yeast

## Abstract

**Background:**

The data-driven inference of intracellular networks is one of the key challenges of computational and systems biology. As suggested by recent works, a simple yet effective approach for reconstructing regulatory networks comprises the following two steps. First, the observed effects induced by directed perturbations are collected in a signed and directed perturbation graph (PG). In a second step, Transitive Reduction (TR) is used to identify and eliminate those edges in the PG that can be explained by paths and are therefore likely to reflect indirect effects.

**Results:**

In this work we introduce novel variants for PG generation and TR, leading to significantly improved performances. The key modifications concern: (i) use of novel statistical criteria for deriving a high-quality PG from experimental data; (ii) the application of local TR which allows only short paths to explain (and remove) a given edge; and (iii) a novel strategy to rank the edges with respect to their confidence. To compare the new methods with existing ones we not only apply them to a recent DREAM network inference challenge but also to a novel and unprecedented synthetic compendium consisting of 30 5000-gene networks simulated with varying biological and measurement error variances resulting in a total of 270 datasets. The benchmarks clearly demonstrate the superior reconstruction performance of the novel PG and TR variants compared to existing approaches. Moreover, the benchmark enabled us to draw some general conclusions. For example, it turns out that local TR restricted to paths with a length of only two is often sufficient or even favorable. We also demonstrate that considering edge weights is highly beneficial for TR whereas consideration of edge signs is of minor importance. We explain these observations from a graph-theoretical perspective and discuss the consequences with respect to a greatly reduced computational demand to conduct TR. Finally, as a realistic application scenario, we use our framework for inferring gene interactions in yeast based on a library of gene expression data measured in mutants with single knockouts of transcription factors. The reconstructed network shows a significant enrichment of known interactions, especially within the 100 most confident (and for experimental validation most relevant) edges.

**Conclusions:**

This paper presents several major achievements. The novel methods introduced herein can be seen as state of the art for inference techniques relying on perturbation graphs and transitive reduction. Another key result of the study is the generation of a new and unprecedented large-scale in silico benchmark dataset accounting for different noise levels and providing a solid basis for unbiased testing of network inference methodologies. Finally, applying our approach to *Saccharomyces cerevisiae* suggested several new gene interactions with high confidence awaiting experimental validation.

## Background

Data-driven inference of intracellular regulatory networks, in particular of those involved in gene regulation, remains to be one key challenge of computational and systems biology.

Many methods for this daunting task have been proposed and new methods are appearing at a high rate [1-6]. The different inference methodologies can be categorized based on the model formalism and the principle used for deriving interactions in a regulatory network: sparse regression [7], correlation-based approaches [8], z-score [9], mutual information [10,11], ANOVA-based analysis [12], Bayesian networks [13], Gaussian graphical models [14], random forest [15], differential equations [16], reaction networks [17] and Boolean networks [18,19]. One final output of all these approaches is the reconstructed network topology, typically given as a (signed or unsigned, directed or undirected) graph. Recent efforts have shown that combining several of the aforementioned methods often outperform all single approaches [6].

It is of utmost importance to rigorously evaluate and compare the large number of methods before one can put confidence in the results of their application. The need for the verification of computational systems biology methods is now recognized worldwide. Notably, the Dialogue on Reverse Engineering Assessment and Methods (DREAM) project organizes international gene regulatory network inference challenges and evaluates the solutions submitted by participating research groups in a transparent manner [6,20,21]. This way a “collaborative-competition” is established in which complicated problems are addressed as a community rather than individual laboratories [22]. Recently, it was demonstrated that such community effort was fruitful for the inference of an improved gene regulatory network of *Escherichia coli* and the inference of a novel gene regulatory network for the bacterium *Staphylococcus aureus*[6].

Verification of inference methods requires benchmark datasets [22]. Benchmarking on real biological data is challenging as true biological networks are largely unknown [23]. The availability of realistically simulated datasets is therefore of utmost importance for the verification of these methods. Only for simulated data can we be certain about the true complex system underlying the data. Simulated data has been used to validate methods, but typically the data was generated with small networks (containing 10–100 genes) [24,25] and with the same models as used by the inference [26,27]. A step to more realistic benchmark data was made in 2003 [28], generating simulated gene expression data using equations based on enzyme kinetics (for use of these data in method evaluations, see e.g. [8,29]). As regulatory network inference methods are typically applied to genome-wide data, a necessary next step is to perform evaluations also on genome-scale.

In this paper we revisit two related gene network inference methods: down-ranking of feed-forward loops (DR-FFL [30]) and TRANSitive reduction for WEighted Signed Digraphs (TRANSWESD [31]). Both approaches were successfully employed (ranked first and third, respectively) in the DREAM4 *In Silico Network 100 nodes* challenge. In this challenge, the task was to reverse engineer gene networks from (simulated) steady-state and time-series data. DR-FFL and TRANSWESD share a common core as they both try to infer a minimal regulatory graph that can explain the gene expression changes observed in perturbation experiments. In particular, both methods apply the principle of *Transitive Reduction* to identify and eliminate edges reflecting indirect effects. Since both DR-FFL and TRANSWESD were ranked high, their underlying inference strategy could provide a generally promising approach for gene network inference.

Network reconstruction based on transitive reduction usually involves three steps of which the last can be seen as optional: 

**Step 1 (Generation of a perturbation graph)**: A *perturbation graph*$G^{P}$ is generated from the perturbation data, i.e., a directed edge from a node *i* to a node *j* (*i* → *j*) is included in $G^{P}$ if a perturbation in *i* changed the level of *j* significantly (significance to be measured by a certain criterion). Sometimes, the edges are also labeled by a sign and might also get a weight indicating their confidence or likelihood.

**Step 2 (Transitive reduction)**: As an edge in the perturbation graph may reflect a direct but also an indirect effect between two nodes, the goal of the second step – the transitive reduction – is to identify and eliminate indirect effects in $G^{P}$ yielding the final reconstructed graph $G^{T}$. As a general rule for transitive reduction, an edge introduced due to indirect effects is detected by searching for alternative paths in $G^{P}$ which could induce the same net effect as this edge. We say that such a path *explains* the edge and the latter is then removed.

**Step 3 (Edge sorting)**: Normally one would consider all edges contained in $G^{T}$ as the true edges. In an optional third step, all edges of the reconstructed graph $G^{T}$ are ranked in a list according to a given confidence score for each edge. For certain applications it might be useful to augment this list also by “edges” (together with their confidence values) not contained in $G^{T}$ (i.e., edges which were not contained in $G^{P}$ or which were removed from the latter when computing the transitive reduction $G^{T}$). In this way we get an ordered list of all potential pairwise interactions according to their confidence score.

These three steps are common to all approaches using transitive reduction (abbreviated by TR in the following) but different variants may arise (i) by using different approaches to derive the perturbation graph (abbreviated PG) in Step 1 or (ii) by considering different criteria a path must fulfill in order to explain a given edge in Step 2, or (iii) by different edge sorting schemes to be used in Step 3. For example, DR-FFL [30] uses a z-score-based strategy to generate the PG and does not consider edge signs in the TR step when searching for valid paths that can explain certain edges. In contrast, TRANSWESD [31] generates the PG by selecting edges that satisfy two related but distinct statistical conditions whereas the actual TR procedure accounts for edge signs and also edge weights when searching for suitable paths that can explain a given edge.

In the present study, we propose and test novel variants for each of the three steps mentioned above, i.e., for PG generation, for TR, and for edge sorting. As one major outcome, we present particular combinations of PG generation and TR strategies which yielded superior results in diverse benchmark tests outperforming by far the two original approaches. As benchmarks we used not only the DREAM4 *In Silico Network* challenge but also a novel and unprecedented synthetic compendium consisting of several realistic 5000-gene networks simulated with varying biological and measurement error variances resulting in a total of 270 datasets. In both benchmarks we focus on perturbations induced by single gene knockouts. Such experiments can be carried out at genome-scale at least in some model organisms (see, for example, [32-35]). As a realistic application scenario we used our framework for inferring gene interactions in yeast *Saccharomyces cerevisiae* based on a library of gene expression data measured in mutants with single knockouts of transcription factors [35]. The reconstructed network shows a significant enrichment of known interactions, especially within the (most relevant) edges identified with highest confidence.

The results of the benchmarks demonstrate the relative performance of the different approaches, moreover, they also enable us to draw some general conclusions. For example, it turns out that when pruning the PG by TR, it is often sufficient or sometimes even favorable to restrict the search on paths having a length of only two. We also demonstrate that edge weights are highly beneficial for TR whereas edge signs are of minor importance (the latter finding was also reported in [36]). We give an explanation for these observations from a graph-theoretical perspective.

## Methods

We start with a brief description of the original TR methods DR-FFL and TRANSWESD which inspired the novel inference algorithms presented herein. Afterwards we introduce the new variants for PG generation, TR, and edge sorting. For the PG generation algorithms, we assume that we are given the following input variables (for a network of *n* genes): 

• a 1 × *n* row vector **G**^*wt*^ containing the (possibly preprocessed) wild-type gene expression data

• the *n* × *n* matrix **G**^*ko*^ containing the (possibly preprocessed) measured steady-state gene expression levels after perturbing/knocking-out each single gene. The element $G_{i,j}^{\text{ko}}$ stores the gene expression level of gene *j* after perturbing gene *i*.

These input variables directly correspond to the datasets provided in the DREAM4 challenge and in our novel compendium of simulated large-scale networks (described below).

### Down-ranking of feed-forward loops (DR-FFL)

The DR-FFL algorithm described in [30] used the following strategies for the three steps:

#### Step 1 (PG generation)

In a preprocessing step, a confidence weight is assigned to each possible edge *i* → *j* of the network by computing the absolute value of the standard z-score *z*_*ij*_. The latter quantifies the difference between the expression $G_{i,j}^{\text{ko}}$ of gene *j* under knockout/perturbation of gene *i* and its mean *μ*_*j*_, normalized by the standard deviation *σ*_*j*_:

(1) $$z_{\text{ij}}=\frac{G_{i,j}^{\text{ko}}-\mu_{j}}{\sigma_{j}}.$$

Mean *μ*_*j*_ and standard deviation *σ*_*j*_ are computed on all available expression measurements of gene *j*, including the wild-type $G_{j}^{\text{wt}}$. Then, the PG $G^{P}$ is obtained by selecting all those edges whose |*z*_*ij*_| is larger than a given threshold *β*. We then denote the PG generated by the original DR-FFL method by PG^1^.

#### Step 2 (TR)

DR-FFL circumvents possible problems arising in TR of cyclic graphs by allowing only those edges to be removed that connect nodes from different strongly connected components (a strongly connected component in a directed graph is a maximal subgraph in which for each ordered pair of nodes a path exists connecting these nodes). DR-FFL uses unsigned and unweighted TR, i.e., an edge *i* → *j* is removed from $G^{P}$ if *i* and *j* are from different components and if there is an alternative path connecting *i* and *j* without using edge *i* → *j*.

#### Step 3 (Edge sorting)

The confidence weights |*z*_*ij*_| of the remaining edges in the graph $G^{T}$ obtained after TR are increased by a constant offset such that all edges in $G^{T}$ are ranked higher than all other potential edges (not contained in $G^{T}$). The latter are listed below the edges of $G^{T}$ according to their confidence weight computed in Step 1 (PG generation).

### TRANSWESD

TRANSWESD (TRANSitive reduction for WEighted Signed Digraphs) was introduced in [31] with the goal to generalize and improve previous TR approaches [37-39] to make it amenable for the reconstruction of large biological networks.

#### Step 1 (PG generation)

TRANSWESD constructs the PG $G^{P}$ via two thresholds: an edge *i* → *j* is introduced in $G^{P}$ if (i) a measure similar to the z-score |*z*_*ij*_| used by DR-FFL exceeds a given threshold *β* and (ii) if the absolute change of the state of node *j* when perturbing *i* exceeds a certain minimal deviation *γ*, i.e. if $|G_{j}^{\text{wt}}-G_{\text{ij}}^{\text{ko}}|>\gamma$. Each edge *i* → *j* gets a sign $s_{\text{ij}}=\text{sign}\left(G_{j}^{\text{wt}}-G_{\text{ij}}^{\text{ko}}\right)$ indicating whether the changes in *i* and *j* have the same direction (positive sign) or not (negative sign). In addition, a weight *w*_*ij*_ is assigned to each edge *i* → *j* quantifying its *uncertainty* or behavioral distance (i.e., a large weight indicates a low confidence of this edge). Accordingly, TRANSWESD uses *w*_*ij*_ = 1 − |*c*_*ij*_| with *c*_*ij*_ being the conditional correlation coefficient between genes *i* and *j* which is computed from all experiments except those where gene *i* was directly perturbed. More specifically, herein we define the conditional correlation coefficient *c*_*ij*_ as the Pearson correlation coefficient computed from all measurements of nodes *i* and *j* (columns in **G**^*wt*^ and **G**^*ko*^) except in the experiments where *j* was knocked-out. The PG generated by the original TRANSWESD procedure is denoted by PG^2^.

#### Step 2 (TR)

A particular feature of TRANSWESD is that it can deal with signed and weighted PGs and that cycles are allowed. The TR rule is as follows: An edge *i* → *j* with sign *s*_*ij*_ and weight *w*_*ij*_ is removed if there is an alternative path *P*_*ij*_ (*i* ⇒ *j*) which connects *i* and *j* and fulfills the following requirements: (i) *P*_*ij*_ is simple, i.e., it does not contain a cycle; (ii) *P*_*ij*_ does not involve edge *i* → *j*; (iii) the overall sign of *P*_*ij*_ (obtained by multiplying the signs of all its edges) is the same as *s*_*ij*_; and (iv) the maximum weight of all edges on path *P*_*ij*_ (denoted by *w*_*max*_(*P*_*ij*_)) fulfills

(2) $$w_{\text{max}}(P_{\text{ij}})<\alpha \cdot w_{\text{ij}}.$$

The confidence factor *α* is typically chosen close to (but smaller than) unity; the default value used by Klamt et al. [31] is 0.95. With *α* < 1 it is ensured that all edges in the path *P*_*ij*_ have a higher confidence than the edge *i* → *j*. However, in some cases it can nevertheless be advantageous to use also *α* > 1. If a path *P*_*ij*_ with the four required properties exists in the PG, then the observed effect of *i* upon *j* is considered to be explained (induced) by path *P*_*ij*_. All edges *i* → *j* in the PG fulfilling these conditions are considered to be (potentially) removable and are collected in a set *R*. If the graph is acyclic, TR is simple and unique and all potentially removable edges in *R* can be deleted immediately. The situation is more complicated in cyclic graphs: the result of TR can become non-unique, depending on the order of edge removals. TRANSWESD uses a reasonable rule to resolve non-uniqueness: it removes the edges of *R* iteratively starting with the highest weight (lowest association) first. As a second problem in cyclic graphs, it may then happen that a formerly removable edge in *R* becomes non-removable because certain paths may have been interrupted by preceding deletions of other removable edges. Even worse, an edge might still potentially be removable but its elimination would lead to the interruption of a path that was required to explain an edge already removed in a previous iteration (see the example below). It is therefore necessary to explicitly test, in each iteration, whether upon removal of the next edge of *R* all edges originally contained in the PG $G^{P}$ are still explainable by the remaining graph (otherwise this edge has to be reinserted). This may require extensive shortest path calculations.

Therefore, to reduce the computational effort in large-scale cyclic graphs, TRANSWESD provides two parameters (*path_exact* and *full_check*) to allow for the (optional) use of approximate solutions which may drastically reduce the required computation time. Since computing the shortest path of a given sign in cyclic signed digraphs is an NP-complete (and thus delicate) problem, starting TRANSWESD with *path_exact=0* enforces the use of approximate path calculation algorithms which have been shown to produce no or only few errors in large-scale biological networks [31,40]. The *full_check=0* option can be used to suppress recomputation of shortest path lengths after deleting an edge (thus assuming that the relevant path lengths will not change). To our experience from numerous tests, there are usually only minor effects on the reconstruction quality when using this simplification. As we deal herein only with large-scale networks, we used *path_exact=full_check=0* in all calculations.

**Figure 1:**
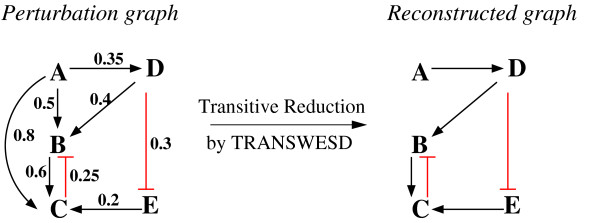
**Example of a perturbation graph and its transitive reduction computed with TRANSWESD.** A given signed and weighted perturbation graph (left) and its transitive reduction as computed by TRANSWESD (right).

We illustrate the approach with the example shown in Figure 1. The graph on the left-hand side displays a hypothetical cyclic PG with its edge weights and signs. Using the standard confidence factor of *α* = 0.95, in principle, three edges could be identified as indirect effects as for each of them a suitable explaining path would exist. This concerns the edge *A* → *C* (explained by path *A* → *B* → *C* and alternatively also by path *A* → *D* → *B* → *C* both fulfilling the sign and weight conditions), the edge *A* → *B* (explained by path *A* → *D*→*B*) and the edge *D* → *B* (explainable by the path *D* ⊣ *E* → *C* ⊣ *B*). These three edges form the set *R* of potentially removable edges. According to the rules, TRANSWESD removes first edge *A* → *C* as it has the largest weight (lowest confidence). In the second iteration, *A* → *B* can be safely removed. Now the algorithm has to stop even though the edge *D* → *B* is still explainable by the path given above. If we removed this edge, no positive path from *A* to *B* and from *A* to *C* would remain in the graph, i.e., the originally observed influence of *A* on *B* and *C* would not be captured anymore. This example shows that TRANSWESD may keep an edge in the graph, even if there is an explaining path for it. The resulting graph $G^{T}$ for this example is shown on the right-hand side of Figure 1. (Note: with *full_check=0* the edge *D* → *B* would be (wrongly) removed in addition to the others whereas *path_exact=0* had no effect).

#### Step 3 (Edge sorting)

TRANSWESD ranks the edges according to their weights computed in Step 1 (PG generation): edges with highest confidence (lowest weights) are placed first. Edges retained in $G^{T}$ are put first followed by edges that were contained in the PG $G^{P}$ (but removed during TR). The last group comprises all other pairwise interactions; their order is also determined by the conditional correlation coefficient *c*_*ij*_.

### Novel variants

DR-FFL and TRANSWESD were successfully applied and highly ranked in the DREAM4 network reconstruction challenge. However, when we compared and mixed both methods (e.g., by replacing Step 1 (PG generation) of TRANSWESD with Step 1 (PG generation) of DR-FFL) we realized that even better approaches, in particular for Step 1 (PG generation) (PG generation) and Step 2 (TR) (TR), might exist. In the following we describe several new variants focusing on those which in the benchmarks performed significantly better than the original DR-FFL and TRANSWESD versions (see Results and discussion section).

#### Perturbation graph

The novel PG generation procedure delivers: 

• The signed and directed PG $G^{P}$ itself.

• A matrix **W**^*t*^ containing the weights of the edges in $G^{P}$ to be used by the transitive reduction algorithm. The element **W**^*t*^(*i*,*j*) contains the weight of the edge *i* → *j* in $G^{P}$; it is set to *∞* if the edge was not included in the PG.

• A matrix **W**^*r*^ containing the confidence weights for all possible interactions (*i*,*j*) to be used in the edge ranking procedure in Step 3. In contrast to **W**^*t*^, this matrix contains a weight for all pairs (*i*,*j*) (except for *i* = *j* as we exclude self-loops), even if *i* → *j* is not contained in $G^{P}$.

A key difference of the novel PG algorithms compared to the strategies used by DR-FFL and TRANSWESD is that different edge weights are used for TR and for edge sorting. Moreover, the selection of candidate edges and the calculation of edge weights are based on (combinations of) correlation and z-score measures. In detail, the following calculations are performed: 

1. Compute the *n* × *n* conditional correlation matrix **C** from the expression measurements **G**^*wt*^ and **G**^*ko*^.

2. Use **G**^*ko*^ to compute the *n* × *n* z-score matrix **Z** comprising the z-score values of all (potential) edges.

3. Compute the *n* × *n* matrix **Z**^*c*^ as the z-score calculated on the absolute value of the entries of the conditional correlation matrix **C**, and add a (minimal) offset to obtain positive values: **Z**^*c*^ > 0.

4. Build the PG by defining the following set of edges: 

• $S^{1}$ comprises all node pairs (*i*,*j*) for which |**Z**_*i*,*j*_| > *β*.

• $S^{2}$ comprises all node pairs (*i*,*j*) for which |**C**_*i*,*j*_| > *γ*.

• $S^{3}$ is the set of all node pairs (*i*,*j*) whose z-score and correlation values have opposite sign: **C**_*i*,*j*_ · **Z**_*i*,*j*_ < 0.

• $B=S^{1}\cap S^{2}\cap S^{3}$ is the set of node pairs (*i*,*j*) satisfying the three previous conditions.

• $Z^{p}$ is the set of node pairs (*i*,*j*) with positive z-score value.

• $Z^{n}$ is the set of node pairs (*i*,*j*) with negative z-score value.

• $E^{p}=Z^{n}\cap B$ is the set of positive edges of the PG.

• $E^{n}=Z^{p}\cap B$ is the set of negative edges of the PG.

• $G^{p}=E^{p}\cup E^{n}=B$ yields the PG.

5. Compute the ranking weight matrix by normalizing **W**^*r*^ = |**Z**| + **Z**^*c*^ between 0 and 1.

6. Compute the weight matrix **W**^*t*^ to be used for transitive reduction in TRANSWESD as **W**^*t*^ = **1** − **Z**^*c*^.

Using this scheme, the PG is built by selecting all edges where (i) the z-score exceeds a given threshold *β*, (ii) the conditional correlation exceeds another threshold *γ*, and (iii) the signs of z-score and conditional correlation are opposite. The latter condition is justified because a positive z-score for the edge (*i*,*j*) is computed when the deletion/decrease of *i* (due to knockout or knockdown) yields an increase in the activity of *j* which should corresponds to a negative correlation between *i* and *j*. The same correspondence exists between negative z-score and positive correlation. Obviously, measurement noise may invalidate the truth of these statements, thus we only keep edges that are consistent with respect to this sign rule. With the rule described above, the positive edges contained in $E^{p}$ stem from a negative z-score and the negative edges contained in $E^{n}$ from a positive z-score. In the following we denote the PG generated by the above procedure PG^new^.

The weights $W_{i,j}^{r}$ used for edge ranking take equally-weighted into account (i) the absolute value of the standard z-score (Equation (1)) of the deviations induced by the perturbation in *i* and (ii) the z-score of the deviations of the conditional correlation between *i* and *j* relative to the averaged conditional correlations related to gene *j*. As far as we know, a z-score of conditional correlations has not yet been used in the context of network inference, however, the ranking weights introduced above proved to be optimal in the benchmarks delivering an edge sorting of high quality. Below we show that the new PG generation approach in combination with the proposed ranking scheme may already deliver a valuable approximation of the network itself but can often be further improved by TR techniques.

Regarding the weights to be used for TR (**W**^*t*^), benchmark tests showed us that it is beneficial to use only the z-score of the conditional correlation coefficients.

#### Transitive reduction

Identifying and pruning edges representing the indirect interactions in $G^{P}$ finally yielding $G^{T}$ is the central goal of transitive reduction. We here present some novel and generalized variants of TR inspired from the original versions of DR-FFL and TRANSWESD.

We observed that the TR used by TRANSWESD (see Step 2 (TR) of TRANSWESD described above) can be generalized in multiple ways: 

• One may consider unweighted TRANSWESD by setting *α* = *∞* in the weight rule (2).

• One may consider unsigned TRANSWESD by setting all edge signs in $G^{P}$ to “+”. In this case, the algorithm becomes simpler (polynomial instead of NP-complete) as the calculation of shortest paths does not need to distinguish between positive and negative paths. It is then, however, still important to keep the weights to avoid non-unique results in cyclic networks.

• When searching for a suitable path *P*_*ij*_ that can explain a certain edge *i* → *j*, one may restrict the search on paths involving not more edges than a predefined number *L*. In this way one would manifest the expectation that observed indirect effects can be traced back to short paths.

With these generalizations we introduce the notation TRANSWESD^S,W,L^ to specify the chosen TR variant: S ∈ {u,s} indicates whether the signed (s) or unsigned (u) TR version is used; W ∈ {u,w} specifies either the unweighted (u) or weighted (w) version; and L specifies the maximal path length allowed. Accordingly, the original TRANSWESD version corresponds to TRANSWESD^s,w,*∞*^. We also observe that TRANSWESD^u,u,*∞*^ mimics TR used by DR-FFL when removal of edges within one and the same component would be blocked.

However, we soon realized that the unweighted variant of TRANSWESD does not perform very well, in particular when combined with the *full_check=0* option (see above). We therefore do not analyze the unweighted version in detail but keep the notation for consistency with respect to the following variant.

In addition to the modified version of TRANSWESD, we introduce a related but different strategy which we call *local transitive reduction* (LTR). There are two key differences: only paths of length 2 are considered as possible explanations for indirect effects and an alternative condition on the edge weight is introduced replacing rule (2). The LTR algorithm considers an edge *i* → *j* *potentially* removable if three criteria are fulfilled: (i) existence of a feed-forward loop, i.e. $\{i\to j,i\to k,k\to j\}\in G^{P}$; (ii) sign consistency, i.e. *s*_*ij*_ = *s*_*ik*_ · *s*_*kj*_; and (iii) the weight condition:

(3) $$\alpha \cdot Z_{\text{ij}}^{c}\leq Z_{\text{ik}}^{c}\cdot Z_{\text{kj}}^{c},\;(\alpha >0).$$

Recall that we introduced **Z**^*c*^ as the z-score of the correlation coefficients and that the relation to the edge weight **W**^*t*^ which we use for modified TRANSWESD is thus simply **Z**^*c*^ = **1** − **W**^*t*^. Therefore, the smaller $Z_{\text{ij}}^{c}$ the higher the confidence that the path *i* → *k* → *j* can explain the edge *i* → *j* (thus, a large weight is here associated with high confidence).

Analogously as described for TRANSWESD, to deal with non-uniqueness, the potentially removable edges are iteratively deleted according to the edge weights (lowest confidence first) and for each edge to be removed it is checked, whether all edges originally contained in $G^{P}$ are still explainable by a 2-path in the remaining graph (otherwise this edge is kept).

Although LTR is also a weighted and signed TR variant, it is considerably simpler than TRANSWESD as it uses a simple triangle rule which is much easier to check than searching for suitable paths. For this reason, in contrast to TRANSWESD, we can easily use the exact variant with *path_exact=full_check=1* in large-scale networks. As will be shown in the section, despite its simplicity, LTR yielded excellent performance in the benchmarks. For LTR we also tested different variants, including the unweighted (condition (3) is dropped by setting *α* = 0), the unsigned and the unsigned/unweighted version (in the latter, only the 2-path *i* → *k* → *j* must exist to render edge *i* → *j* removable, irrespective of edge signs and weights). We introduce a similar notation as for TRANSWESD: LTR^S,W^ indicates whether edge signs (S ∈ {u,s}) and weights (W ∈ {u,w}) are considered or not; the length parameter *L* becomes obsolete as it is fixed to 2.

#### Edge sorting

We use a simple edge ranking procedure which is similar to the strategy used by DR-FFL and (original) TRANSWESD. Note that all *n*(*n* − 1) potential edges (except self-loops) are included into this list, also those that were not contained in the PG $G^{P}$ or that were removed during TR. The position of each edge is determined by the ranking weights stored in **W**^*r*^ (see above): edges with highest ranking weights are put first. To ensure that edges contained in the final graph $G^{T}$ are really ranked higher than all other edges, an offset is added to the weight of all edges in $G^{T}$.

## Results and discussion

In the following we present performance results of the new PG generation algorithm in combination with the modified TRANSWESD and the new LTR technique for subsequent transitive reduction. We used two different case studies for benchmarking: (i) the datasets of the DREAM4 *InSilico_Size100* network inference challenge, and (ii) a novel large-scale synthetic compendium consisting of 30 5000-gene networks simulated by SysGenSIM [41] with different connectivities and noise levels. The DREAM4 benchmark also enables a comparison of the performances of the new approaches with its inspiring original techniques DR-FFL and (old) TRANSWESD. Generally, in the case of in silico datasets (with known gold standard), the goodness of the predictions is evaluated based on the established Area Under the Curve (AUC) measures of ROC (Receiver Operating Characteristic) and PR (Precision-Recall) curves. The AUPR is the most informative (and the only shown) performance measure for the case studies in this paper due to the sparsity of gene networks implying large AUROC values differing only insignificantly for the different methods.

In addition to the two in silico studies where the reconstruction results can be evaluated by a perfect gold standard, we used our framework in a realistic application scenario to infer gene interactions in yeast *Saccharomyces cerevisiae* based on a library of gene expression data measured in mutants with single knockouts of transcription factors.

### Performance on DREAM4 networks

In the DREAM4 *InSilico_Size100* network reconstruction challenge [21,42], simulated steady-state measurements of the expression of each gene in the wild-type as well as in the single-gene knockout and single-gene knockdown mutant were provided for 5 different in silico networks (100 nodes each) from which the networks had to be reconstructed. We only make use of wildtype and knockout data as they directly support the generation of the PG (knockdown data can, in principle, further improve the results; see below). For assessing the quality of reconstructed networks, an evaluation script is available at the DREAM website [43] which computes an overall score obtained from the geometric mean of p-values calculated for the AUPR and the AUROC measures from all 5 reconstructed networks.

We considered predictions by several combinations of the original as well as of the new PG generation and TR methods. The methods’ parameters were chosen according to previously used values (e.g., *α*) or according to preliminary tests. Importantly, one and the same parameter set was used for all five networks, i.e., no optimization was conducted for every single network. The DREAM4 evaluation script was used to compute the respective overall scores which are summarized in Table 1.

**Table 1:** Performance of the inference algorithms on the DREAM4 networks (100 nodes)

**DREAM4 best performers**				**Score**					
**Team 395 (PG**^**1**^** + DR-FFL)**				**71.5889**					
Team 296				71.2970					
**Team 515 (PG**^**2∗**^** + TRANSWESD**^**s,w,∞**^**)**				**64.7150**					
Team 466				63.4060					
Team 549				63.1050					
Inference algorithm	*β*	*γ*	*α*	Score	Edges	TPs	FPs	FNs	TP^100^
PG^1^	2.00	-	-	70.3495	349.4	103.4	246.0	101.4	58.0
PG^1^ + DR-FFL	-	-	-	71.5889	267.4	83.6	183.8	121.2	62.4
**PG**^**1**^** + TRANSWESD**^**u,w,∞**^	**-**	**-**	**0.95**	**73.0444**	**225.0**	**97.4**	**127.6**	**107.4**	**60.8**
PG^1^ + TRANSWESD^u,w,2^	-	-	1.50	79.6644	153.8	83.0	70.8	121.8	70.4
**PG**^**1**^** + LTR**^**u,u**^	**-**	**-**	**-**	**79.7428**	**261.2**	**92.4**	**168.8**	**112.4**	**72.2**
PG^1^ + LTR^u,w^	-	-	0.15	79.1609	262.0	92.8	169.2	112.0	72.2
PG^2^	2.60	0.05	-	65.8012	398.2	98.0	300.2	106.8	58.2
PG^2^ + DR-FFL	-	-	-	64.2614	372.8	94.4	278.4	110.4	58.0
PG^2^ + TRANSWESD^u,w,*∞*^	-	-	0.95	65.6504	256.6	86.0	170.6	118.8	64.4
PG^2^ + TRANSWESD^s,w,*∞*^	-	-	0.95	66.0970	260.8	86.8	174.0	118.0	66.6
PG^2^ + TRANSWESD^u,w,2^	-	-	1.50	66.5562	224.0	81.0	143.0	123.8	64.2
**PG**^**2**^** + TRANSWESD**^**s,w,2**^	**-**	**-**	**1.50**	**68.1534**	**249.2**	**84.4**	**164.8**	**120.4**	**67.0**
PG^2^ + LTR^u,u^	-	-	-	65.4214	253.0	82.2	170.8	122.6	64.6
PG^2^ + LTR^s,u^	-	-	-	67.7407	274.0	86.4	187.6	118.4	66.6
PG^2^ + LTR^u,w^	-	-	0.15	67.2567	271.4	85.6	185.8	119.2	66.0
**PG**^**2**^** + LTR**^**s,w**^	**-**	**-**	**0.15**	**68.5959**	**288.2**	**88.4**	**199.8**	**116.4**	**67.2**
**PG**^**new**^	**2.00**	**0.00**	**-**	**81.7594**	**250.2**	**99.6**	**150.6**	**105.2**	**66.6**
PG^new^ + DR-FFL	-	-	-	80.3085	179.8	82.0	97.8	122.8	66.2
PG^new^ + TRANSWESD^u,w,*∞*^	-	-	0.95	85.3288	179.2	90.2	89.0	114.6	72.0
PG^new^ + TRANSWESD^s,w,*∞*^	-	-	0.95	85.7898	183.0	92.0	91.0	112.8	72.8
PG^new^ + TRANSWESD^u,w,2^	-	-	1.50	88.0570	147.6	86.0	61.6	118.8	72.6
**PG**^**new**^** + TRANSWESD**^**s,w,2**^	**-**	**-**	**1.50**	**88.5728**	**150.4**	**87.8**	**62.6**	**117.0**	**72.8**
PG^new^ + LTR^u,u^	-	-	-	88.2217	166.8	91.8	75.0	113.0	74.2
PG^new^ + LTR^s,u^	-	-	-	88.6350	169.4	93.4	76.0	111.4	75.2
PG^new^ + LTR^u,w^	-	-	0.15	88.5203	168.4	92.8	75.6	112.0	75.0
**PG**^**new**^** + LTR**^**s,w**^	**-**	**-**	**0.15**	**88.8005**	**169.8**	**93.8**	**76.0**	**111.0**	**75.8**

The table summarizes the performance (overall score, true positives (TPs), false positives (FPs) and false negatives (FNs)) of the different PG generation and TR algorithms when applied to the DREAM4 networks together with the displayed (optimal) parameters. The last column TP^100^ shows the average number of TPs within the first 100 top-ranked (reconstructed) edges. As a comparison, the scores of the 5 best-performing algorithms within the challenge are shown. PG^2∗^ denotes PG^2^ computed with a minor bug in the original implementation.

We recall that the combined use of the unsigned and z-score-based PG^1^ with DR-FFL [30] originally obtained the best score (71.59) for the DREAM4 challenge, while the coupling of PG^2^ and (original) TRANSWESD was ranked third with a score of 64.71 (see [31]). Although we used here only the knockout data (in [31] both knockout and knockdown data were used for computing the correlation coefficents) the results presented in Table 1 are slightly better (66.10) by fixing a small bug in the computation of PG^2^.

The DREAM4 best overall score of 71.59 is already exceeded by just applying unsigned TRANSWESD^u,w,*∞*^ or unsigned and unweighted LTR^u,u^ to the unsigned perturbation graph PG^1^. This supports the statement in [44] about the weakness of the original DR-FFL algorithm, where transitive reduction is applied only to edges between but not within strongly connected components of the PG. In fact, TRANSWESD^u,w,*∞*^ and especially TRANSWESD^u,w,2^ and LTR^u,u/w^ improve the score of PG^1^ significantly up to 79.74.

Regarding the results for PG^2^ originally used by the TRANSWESD method in [31] we observe that the quality (score) of the PG is lower than for the simple z-score PG^1^. Although all tested TR techniques (except DR-FFL) can improve the score, it remains below the performance results obtained for the z-score approach PG^1^.

**Figure 2:**
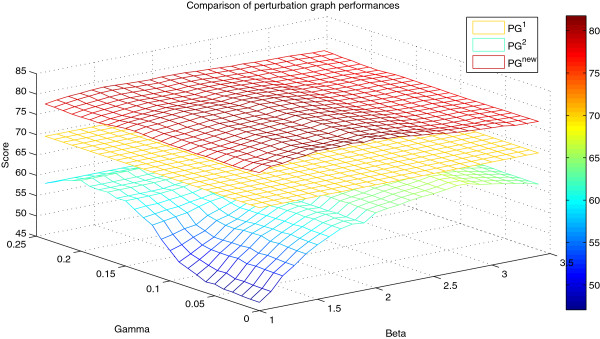
**Performance of the novel perturbation graph PG**^**new**^** in DREAM4 networks (100 nodes).** The overall score of PG^new^ is consistently higher than the scores achieved by the DREAM4 winning submission (71.59, not shown), by PG^1^ (70.35; does not depend on any parameter), and by PG^2^ for the complete space of meaningful parameter values (*β*,*γ*).

Next we tested the performance results of the TR techniques in PG^new^ where we also applied the new edge sorting scheme and sorting weights. As a first observation, a notable quality improvement is obtained by the novel PG^new^ alone achieving a score of 81.76 which is markedly higher than the scores obtained by PG^1^ and PG^2^, even after TR. A somewhat unexpected result was that the *γ* threshold for the conditional correlation coefficients was virtually not required as its optimal value turned out to be 0. However, in other tests described below, using a non-zero value for this threshold in combination with *β* (for the z-score) turns out to be beneficial. We also analyzed the robustness of the quality of PG^new^ and its edge ordering with respect to the chosen threshold parameters *β* and *γ*: Figure 2 displays the overall score of PG^new^ when varying the threshold parameters showing that it is (i) higher than the previous winning score (71.59), (ii) higher than PG^1^, and (iii) higher than PG^2^ – even for the complete space of meaningful parameter values scanned. Hence, a reasonable robustness of the quality of PG^new^ with respect to the two threshold parameters can be concluded.

We then applied the TR techniques to PG^new^ which increase the scores up to 88.80, thus well above the best score recorded at the DREAM4 challenge. Regarding the different TRANSWESD variants, we see that the signed version (85.79) is only slightly better than the unsigned variant (85.33) whereas “local” TRANSWESD, which takes only paths of length 2 into account, results in a further significant improvement of the score (88.57). In line with these observations, unsigned and signed LTR differ only marginally whereas signed and weighted LTR^S,W^ produces the best overall results, not far from the unweighted variant. Recall that TRANSWESD^s,w,2^ and LTR^S,W^ differ essentially only by condition (2) vs. (3). Although the results of both local variants are comparable, it seems that the rule used by LTR can better predict true indirect effects. Generally, Table 1 shows that all TR techniques work well by strongly decreasing the number of false positive edges (FPs) with only a slight decrease in number of true positives (TPs). Apparently, the best ratio is obtained by local TR variants (i.e., by LTR and TRANSWESD^s,w,2^). We also noticed that the (average) enrichment of TPs under the first 100 reconstructed edges in the sorted edge list (column TP^100^ in Table 1) is especially large confirming the potential of our methods: it reaches 66.6 for PG^new^, 72.8 for TRANSWESD^s,w,2^ and even 75.8 for LTR^S,W^. Hence, there is a high probability that top-ranked edges correspond to true interactions – a desirable property when validating the edges experimentally.

**Figure 3:**
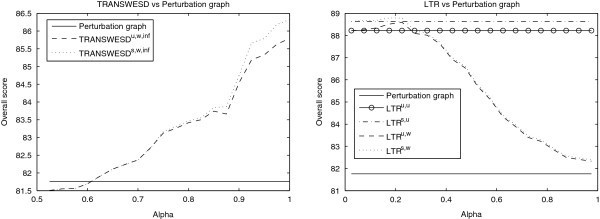
**Robustness of TR variants of TRANSWESD (left) and LTR (right) in DREAM4 networks.** The overall scores achieved after TR with TRANSWESD^·,w,*∞*^ or LTR^·,·^ are consistently higher than the score obtained by PG^new^ for a large range of meaningful values of the confidence factor *α*. TRANSWESD^·,·,2^ was not included because of its different operating range of values.

Figure 3 demonstrates that TRANSWESD (left) and LTR (right) are also fairly robust with respect to the confidence factor, as the score of the perturbation graph PG^new^ is highly improved by both methods for a broad range of meaningful values of *α*. In this context it is also of interest that unweighted LTR yielded very good predictions even without the need to specify any further parameter (as necessary for weighted LTR and TRANSWESD).

We summarize that the best-ranked algorithms of the DREAM4 competition are significantly outperformed by our new methods for PG generation, TR, and edge sorting. We noticed that also other recently published network inference techniques applied to the DREAM4 networks [36,45] reported lower predictions. For example, the highest overall score in [45] is 81.10 obtained by using both knockout and knockdown datasets whereas a score of 73.33 was achieved in [36] by CUTTER-W, an approach similar to unsigned (and weighted) TRANSWESD. Moreover, if we also include knockdown data in our analysis (for calculating the conditional correlation coefficients), the scores in Table 1 grow up by approximately 3–4 points each, reaching a top of 92.03 with PG^new^ + LTR^S,W^. As expected, this confirms that an increase of the number of measurements corresponds to an improvement of the prediction. However, most of the information is already provided by the knockout experiments.

### Performance on SysGenSIM datasets

In order to provide an even more exhausting and more realistic test scenario for the developed inference algorithms, the software SysGenSIM [41] was used to create a new collection of synthetic gene networks and to simulate knockout experiments under different connectivities and noise conditions. SysGenSIM is able to generate large networks with a topology similar to those observed in real organisms, i.e., with a modular structure featuring exponential and power-law behavior for the in- and out-degree distributions of nodes [46]. The generated 30 in silico networks have a considerable (close to genome-scale) size of 5000 nodes each. One third of them has a low average degree (about 7500 edges, i.e. *K* ≃ 1.5), 10 networks have an intermediate average degree (about 10000 edges, i.e. *K* ≃ 2), while the last third of the networks exhibits the largest average degree (about 12500 edges, i.e. *K* ≃ 2.5). Finally, using equations of biochemical kinetics where the degradation rate of gene expression is represented by a first-order process and where the transcription rate exhibits essential features of cooperativity and saturation [28], single knockout experiments have been simulated for all the genes of each network with SysGenSIM’s default kinetic parameters under 9 different combinations of noise conditions (for technical details see [41]). In fact, SysGenSIM allows for the selection of the standard deviation *σ*_*θ*_ of the Gaussian distribution from which the biological synthesis and degradation variances are sampled (parameters *θ*^syn^ and *θ*^deg^ in Equation (1) in [41]) as well as the standard deviation *σ*_*ν*_ of the Gaussian distribution from which the experimental noise *ν* is sampled. As possible values for both standard deviations we considered {0.025,0.05,0.1}, yielding a total of 9 combinations summarized in Table 2. Therefore a grand total of 270 different networks (30 topologies with 9 different noise configurations) with simulations of single-knockout experiments have been produced, the goal being the testing of the inference methodologies under different conditions of edge density, biological variance, and multiplicative measurement noise.

**Table 2:** Noise configurations in simulated datasets

**Configuration**	**Label**	***σ***_***θ***_	***σ***_***ν***_
1	LL	0.025	0.025
2	LM	0.025	0.050
3	LH	0.025	0.100
4	ML	0.050	0.025
5	MM	0.050	0.050
6	MH	0.050	0.100
7	HL	0.100	0.025
8	HM	0.100	0.050
9	HH	0.100	0.100

Columns *σ*_*θ*_ and *σ*_*ν*_ show the values of the standard deviations of the Gaussian distributions with *μ* = 1 from which the biological variances *θ*^syn^ and *θ*^deg^ and the measurement error *ν* were sampled in SysGenSIM. Each configuration is also represented by a 2-character string, indicating the intensity levels (low (L), medium (M), high (H)) of the biological variance (first letter) and of the measurement error (second letter), respectively.

**Figure 4:**
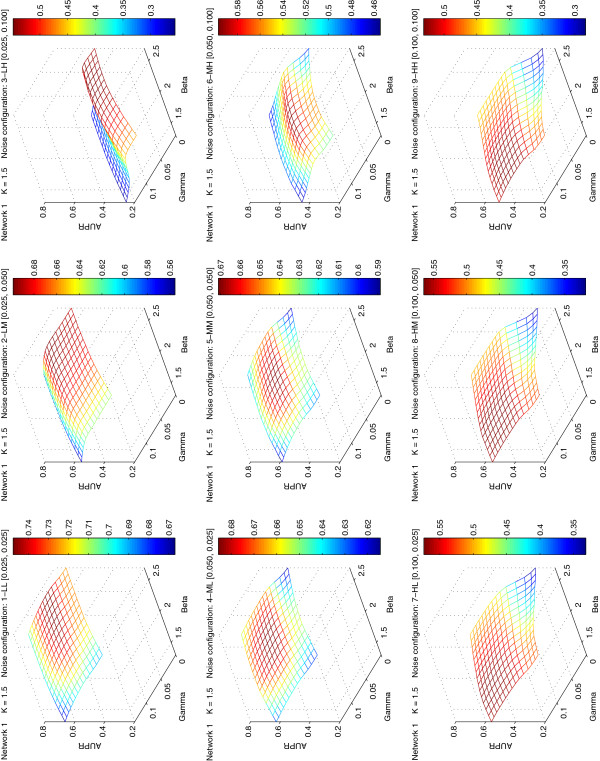
**Performance and robustness of the perturbation graph methodology PG**^**new**^** applied to the 9 noise configurations of network 1 in the SysGenSIM dataset.** The AUPR scores of PG^new^ are fairly robust for a large range of meaningful parameter values *β* and *γ*. The picture shows the performance of the inference of Network 1 (containing about 7500 edges) with respect to the 9 different noise conditions. Similar behaviors can be seen for the other networks (see Figures F1 and F2 in Additional file 1).

Due to the superior performance of PG^new^ we present results only for this PG. Figure 4 displays the performance of PG^new^ for the 9 different noise configurations of network 1 (connectivity *K* ≃ 1.5) in dependency of a wide range of (*β*,*γ*) parameters (examples for *K* ≃ 2 and *K* ≃ 2.5 are shown in Figures F1 and F2 in Additional file 1). The novel PG generation algorithm exhibits reasonable robustness with respect to both noise and threshold parameters. In fact it works decently with the same *β* and *γ* as used for the DREAM4 networks (for the optimal value, *γ* needs to be slightly raised from 0.00 to 0.05), while the procedures for PG^1^ and PG^2^ would need a more extensive re-tuning of the parameters to obtain reasonable results (not shown).

**Figure 5:**
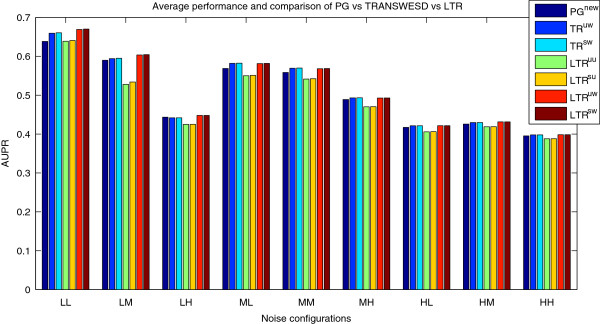
**Performance of the new TRANSWESD and LTR variants on the SysGenSIM dataset.** Parameters used to obtain the perturbation graph were *β* = 2.0 and *γ* = 0.05, while *α* = 0.95 and *α* = 0.15 were selected for the TRANSWESD and LTR variants, respectively. AUPR scores are averaged across the 30 networks (10 networks for each of the three averaged node degrees considered) simulated with the same noise configuration.

The effect of the TR algorithms applied to PG^new^ (Tables 3 and 4, Figure 5) becomes more heterogeneous and differentiated compared to the DREAM4 networks. First of all, we observe that the unweighted versions of LTR decrease in all cases the quality of the perturbation graph PG^new^ whereas weighted LTR and (non-local versions of) TRANSWESD improve it – partially significantly – in all scenarios (with one minor exception). This demonstrates that weighted TR can be highly beneficial. However, local TRANSWESD^s,w,2^, which was comparable with LTR in the DREAM4 networks, achieves similar unfavorable results for these large and noisy networks as unweighted LTR. This confirms again that rule (3) seems to be better suited for *local* TR than rule (2). Furthermore, the quality of the PG as well as the relative improvement by the (weighted) TR techniques depends substantially on the magnitude of the noise level both with respect to AUPR and in the number of TPs and FPs. An interesting observation can be made regarding the effect of biological variance on the reconstruction quality: it appears that moderately increased (medium) biological noise is advantageous in case of high measurement noise for all *K*’s (i.e., networks with noise configuration MH perform better than those with LH; see Figure 5 and Table 3 as well as Tables T1 and T2 and Figure F3 in Additional file 1). Thus, higher biological noise may help to uncover true perturbation effects under high uncertainty of measurements.

**Table 3:** Performance of the inference algorithms on the SysGenSIM networks

	**Noise configuration**								
**Inference algorithm**	**1 - LL**	**2 - LM**	**3 - LH**	**4 - ML**	**5 - MM**	**6 - MH**	**7 - HL**	**8 - HM**	**9 - HH**
PG^new^	0.7388	0.6835	0.5159	0.6735	0.6622	0.5850	0.5141	0.5218	0.4835
PG^new^ + TRANSWESD^u,w,*∞*^	0.7695	0.6906	0.5141	0.6921	0.6778	0.5921	0.5192	0.5269	0.4868
PG^new^ + TRANSWESD^s,w,*∞*^	0.7702	0.6910	0.5142	0.6923	0.6780	0.5922	0.5192	0.5269	0.4868
PG^new^ + TRANSWESD^u,w,2^	0.7335	0.5825	0.5041	0.6471	0.6410	0.5650	0.4963	0.5115	0.4737
PG^new^ + TRANSWESD^s,w,2^	0.7354	0.5929	0.5042	0.6478	0.6417	0.5653	0.4965	0.5116	0.4739
PG^new^ + LTR^u,u^	0.7561	0.6320	0.5114	0.6701	0.6583	0.5783	0.5093	0.5209	0.4816
PG^new^ + LTR^s,u^	0.7570	0.6390	0.5115	0.6705	0.6587	0.5784	0.5094	0.5210	0.4818
PG^new^ + LTR^u,w^	0.7737	0.7051	0.5285	0.6898	0.6751	0.5924	0.5196	0.5291	0.4880
PG^new^ + LTR^S,W^	0.7742	0.7057	0.5285	0.6900	0.6753	0.5925	0.5196	0.5291	0.4880

Each score is the mean of the AUPR computed for the 10 networks with *K* ≃ 1.5 simulated according to the same noise configuration. Thresholds used by the inference algorithms are *β* = 2.0 and *γ* = 0.05 for generating PG^new^, *α* = 0.95 for TRANSWESD^·,w,*∞*^, *α* = 1.50 for TRANSWESD^·,w,2^ and *α* = 0.15 for LTR^·,w^. Analogous performances for *K* ≃ 2 and *K* ≃ 2.5 are shown in Tables T1 and T2 in Additional file 1.

**Table 4:** Statistics on edges from inferred SysGenSIM networks

	***K* ≃ 1.5**			***K* ≃ 2**			***K* ≃ 2.5**		
**Inference algorithm**	**Edges**	**TPs**	**FPs**	**Edges**	**TPs**	**FPs**	**Edges**	**TPs**	**FPs**
PG^new^	14353	6239	8114	20477	7422	13055	27496	8258	19239
PG^new^ + TRANSWESD^u,w,*∞*^	-15.60%	-0.57%	-27.16%	-15.71%	-1.96%	-23.52%	-14.89%	-3.48%	-19.79%
PG^new^ + TRANSWESD^s,w,*∞*^	-15.35%	-0.40%	-26.83%	-15.17%	-1.60%	-22.89%	-14.44%	-3.04%	-19.34%
PG^new^ + TRANSWESD^u,w,2^	-37.47%	-16.01%	-53.97%	-42.82%	-25.03%	-52.93%	-46.69%	-33.13%	-52.51%
PG^new^ + TRANSWESD^s,w,2^	-37.24%	-15.65%	-53.85%	-42.38%	-24.17%	-52.73%	-46.09%	-31.77%	-52.23%
PG^new^ + LTR^u,u^	-32.06%	-9.68%	-49.27%	-35.28%	-15.07%	-46.77%	-37.67%	-19.83%	-45.33%
PG^new^ + LTR^s,u^	-31.93%	-9.51%	-49.17%	-35.03%	-14.61%	-46.64%	-37.36%	-19.20%	-45.16%
PG^new^ + LTR^u,w^	-29.74%	-5.76%	-48.18%	-30.64%	-7.79%	-43.63%	-28.62%	-8.87%	-37.10%
PG^new^ + LTR^S,W^	-29.64%	-5.66%	-48.09%	-30.49%	-7.53%	-43.54%	-28.45%	-8.53%	-37.00%

The number of edges in the perturbation graph and the number of FPs and TPs are shown in the table. The relative reduction of these measures (edges, TPs and FPs) in the graphs obtained after applying the different TR techniques compared to the PG is also displayed. These averaged statistics are computed from the analysis of the graphs obtained after inferring the 30 SysGenSIM networks simulated according to noise configuration 1 (LL; see Table 2). Thresholds used are *β* = 2.0 and *γ* = 0.05 for generating PG^new^, *α* = 0.95 for TRANSWESD^·,w,*∞*^, *α* = 1.50 for TRANSWESD^·,w,2^ and *α* = 0.15 for LTR^·,w^. Analogous tables for the other 8 noise configurations (2, …, 9) are shown in Tables T3–T10 in Additional file 1.

It can also be noticed that, in general, TRANSWESD^s,w, *∞*^ and LTR^S,W^ achieve similar superior AUPR performance, but by different means as manifested in Table 4: the LTR technique prunes the edges of the PG more generously than TRANSWESD^s,w,*∞*^, resulting in a better reduction of false positive edges, but at the same time in an undesired higher decrease of true positives. We can also confirm a result from the DREAM4 benchmark: signed (weighted) LTR and TRANSWESD achieved always better AUPR scores than their unsigned versions (except in one case), but only to a very small extent. This important observation is discussed in more detail in the Conclusion section.

Finally, Figure F3 in Additional file 1 shows how the precision of PG^new^ and the effectiveness of TR decrease when the network connectivity (average node degree *K*) increases. Moreover, the superiority of weighted vs. unweighted TR can again clearly be seen.

### Application to a realistic yeast knockout dataset

The ultimate test for our reverse-engineering algorithm would be the application to a genome-scale real-world dataset of single-gene perturbation experiments (e.g., through single gene knockouts). Only few such datasets are available. The most suitable for our purpose is the *S. cerevisiae* transcription factor knockout expression compendium of Hu et al. [34] where the expression of *n* = 6253 genes was measured after single knockouts (or knockdowns) of *m* = 269 transcription factors (TFs) being the most important regulators in yeast. Herein we refer to the revised dataset provided by Reimand et al. [35] where the original raw data of Hu et al. were reanalyzed with more sophisticated statistical techniques of the BioConductor package [47] leading to an increased informative content of the microarray measurements.

The processed data of Reimand et al. ( http://www.ebi.ac.uk/arrayexpress/experiments/E-MTAB-109) consists of three matrices of size *m* × *n*: 

• **L** contains the log-fold change values for all genes across all knockout experiments.

• **P** includes the p-values for differential expression.

• **A** is the signed adjacency matrix of the graph reconstructed by Reimand et al. The entries *A*(*i*,*j*) correspond to (inferred) edges with a p-value of *P*(*i*,*j*) < 0.05.

Our goal was to re-process the log-fold change values in order to apply our network inference algorithm and to produce a ranked list of edges. For comparing our reconstructed network with the predicted network of Reimand et al. we need a gold standard. However, as a reliable gold standard for gene regulation in *S. cerevisiae* is still not available (otherwise we would not need our inference methods), we made use of four published “silver standard” networks containing experimentally validated interactions (between TFs or from TFs to other genes): 

1. **SS**_1_: is a collection of found chip-chip results and motifs from 162 TFs [48] (size of silver standard network: 162 TFs × 6253 genes).

2. **SS**_2_: is a subset of the binding sites from **SS**_1_ which are also in nucleosome-depleted regions [48] (size of silver standard network: 159 TFs × 6253 genes).

3. **SS**_3_: this silver standard network contains known regulatory interactions between 142 TFs and 3459 targets compiled from the results of genetic, biochemical and ChIP (chromatin immunoprecipitation)-chip experiments [49] (size of silver standard network: 142 TFs × 3459 genes).

4. **SS**_4_: contains interactions between 114 TFs and 5667 targets. This network was used as a reference network for a sub-challenge of the DREAM5 competition [6] (size of silver standard network: 114 TFs × 5667 genes).

In order to obtain a gene expression matrix **G** exploitable by our inference algorithm, we “inverted” the log-fold change values to obtain *G*(*i*,*j*) = 2^*L*(*i*,*j*)^ for all the possible edges. In this way, expression values larger than 1 represent an increase of the gene expression of *j* after the knockout of *i* (i.e., *i* is a inhibitor of *j*), and vice versa a positive regulation for values smaller than 1.

As for the synthetic datasets, the gene expression matrix **G** served then as input to produce the perturbation graph PG^new^ and the weight matrices **W**^*r*^ and **W**^*t*^. The transitive reduction techniques TRANSWESD and LTR were then applied to PG^new^ and the resulting edges for each method were sorted according to our ranking scheme. This sorted edge list was delivered as output (prediction) of our procedure. We performed the whole inference process by employing the same parameters used for the simulated networks, i.e. *β* = 2.00, *γ* = 0.05, *α* = 0.95 for TRANSWESD, *α* = 1.50 for local TRANSWESD, and *α* = 0.15 for LTR. Note that *m* = 269 TFs were perturbed, hence, we can only infer edges that will start in a TF node and point to TF or non-TF genes.

A (predicted) confidence-sorted edge list was also obtained for the original dataset of Reimand et al. by re-sorting the absolute values of the log-fold changes in **L** according to the adjacency matrix **A**, which is then used as a reference to assess the performance of Reimand’s reconstructed network.

Afterwards we evaluated all predictions against the 4 silver standards. To allow for a fair scoring, only common nodes from silver standard networks and prediction lists were taken into consideration, i.e. if edge (*i*,*j*) is in the prediction list but node *i* or *j* is not contained in the silver standard, then the edge is not scored. On the other hand, if node *k* belongs to the silver standard but was not included in the microarray dataset, then all ingoing and outgoing edges of *k* were removed from the silver standard. Accordingly, the size of the silver standard SS_3_ reduced from 142 × 3459 to 122 × 3444 and of SS_4_ from 114 × 5667 to 108 × 5469.

**Figure 6:**
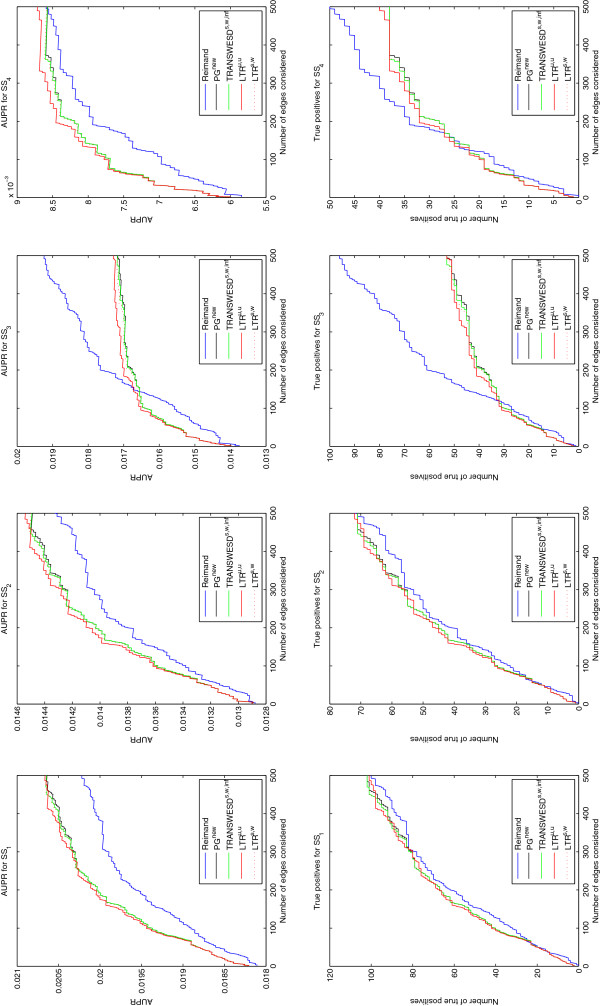
**Performance of the novel inference techniques on the** ***S. cerevisiae* dataset validated against four silver standards.** The plots show the AUPR and the number of true positive edges computed for the 500 best-ranked edges against four “silver standard” networks (see text for explanations). Parameters used to infer the networks are *β* = 2.0 and *γ* = 0.05 for the PG, *α* = 0.95 for TRANSWESD and *α* = 0.15 for LTR.

For each of the four silver standards, Figure 6 shows the AUPR and the number of true positive edges (TP) computed for an increasing number of edges selected from top of the ordered edge lists as given by (1) Reimand’s predictions, (2) the perturbation graph PG^new^, (3) TRANSWESD^s,w,*∞*^, (4) LTR^u,u^, and (5) LTR^S,W^. Generally, the results of our methods (2)-(5) with respect to the four different silver standard networks appear to be satisfactory, though with different measure for the four silver standard networks. It is apparent that our methods work especially well within the 100 – 200 top-ranked edges where all of the inferred networks (2)-(5) show better agreement with the silver standards than the interactions found by Reimand. The PG itself performs again reasonably well and better than Reimand’s network in this region. All variants of TR show positive effects but not among the top-ranked edges because these are immune against pruning (accordingly, for these edges, the results for PG^new^ and for the TR methods are identical). We observe that unweighted and unsigned LTR^u,u^ performed best for this dataset. However, one should keep in mind that no tuning or adaptation of the parameters has been performed which could have prevented a better result for the weighted versions.

When increasing the number of considered edges to 500, it appears that Reimand’s network becomes better and better eventually, in some cases, getting higher overall agreement with the silver standards than our methods. However, we argue that for practical applications (e.g. validation of edge candidates) the first ≈ 100 edges are the most important ones. In this region, given the silver standards, our approach seems to work most efficient yielding high statistical significance: for the four silver standard networks we obtained [42, 27, 31, 20] TPs in the network reconstructed with LTR^u,u^ yielding corresponding p-values of [6.31 · 10^−46^, 6.68 · 10^−28^, 3.93 · 10^−33^, 6.79 · 10^−25^], based on the hypergeometric distribution. These values are very similar for the other four PG/TR-based methods. It is most likely that the number of TPs is even larger given the high probability that not all interactions might be contained in the silver standards. Our prediction list might thus provide useful targets for validations. A sorted list of the 300 (identified) edges with highest confidence and a comparison with the four silver standards can be found in Table T4 in the Additional file 2.

## Conclusion

We presented novel algorithms for the inference of gene regulatory networks from systematic perturbation experiments. These algorithms support the reconstruction of regulatory networks via three steps: (i) PG generation, (ii) TR to remove edges representing indirect effects in the PG, and (iii) sorting of edge candidates. We presented new variants for all of these three steps whose combined use yielded superior results over previous methods when tested with standardized benchmark scenarios.

Regarding the PG, it proved advantageous to identify, weight and sort candidate edges by a mixture of two measures, one being the standard z-score of deviations, the other one the z-score of conditional correlation coefficients. In particular, the latter was highly informative for edge pruning by TR whereas a combined weight of both z-scores proved beneficial for edge sorting. With the new candidate edge selection and edge sorting schemes, we observed that the PG alone (without TR) achieved a reconstruction quality that is far above the results of previous methods *after* TR. Importantly, the quality of the PG appeared to be robust against larger variations in the two required threshold parameters. In this regard, one aspect for future work is to develop algorithms for automatic thresholding, that is to estimate the threshold parameters from the data.

**Figure 7:**
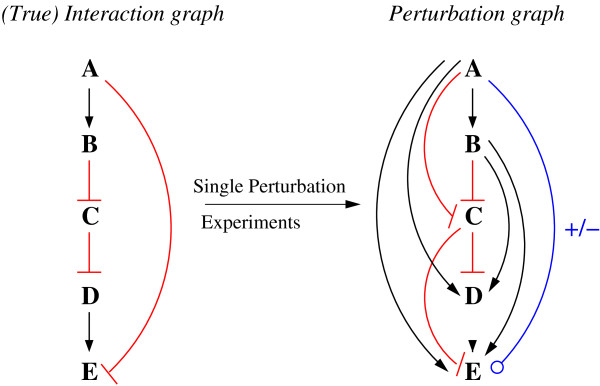
**Example of a (true) graph and its (perfect) perturbation graph representing the transitive closure.** An interaction graph (left) and its (expected) perturbation graph which forms the transitive closure of the original graph (right).

We proposed new variants of TR and, based on unbiased in silico benchmarks, compared them with the original versions of the algorithms. Several key observations could be made: 

1. The DR-FFL method [30] was inferior to all other TR methods tested which led us to the conclusion that TR should be employed not only between but also within cyclic structures. The winning performance of the original DR-FFL in the DREAM challenge can mainly be attributed to its PG which is in parts similar to the one used by PG^new^.

2. We found that explicitly accounting for edge signs almost always improves the results in terms of AUPR but only to a very minor extent. While this is in agreement with the observations made in [36], we give here an extended explanation for this unexpected result. Generally, neglecting the edge sign can only be “harmful” during TR, if the true network contains a negative feed-forward loop (FFL). As an example, Figure 7 (left) shows a hypothetical interaction graph containing one such negative FFL between node A and E (consisting of a positive path and a negative edge from A to E). If we now assume that all nodes are perturbed in single perturbation experiments we would get, in the ideal case, a PG as shown in Figure 7 (right; weights not considered here). This PG corresponds to the *transitive closure* of the original graph, in which each node *i* induces a significant effect on another node *j* if there is an edge or path from *i* to *j* in the true graph. What we can now see is that each edge contained in the PG but not in the true graph (reflecting thus an indirect effect) is part of a positive FFL consisting of this edge and a path of the same sign. This happens because all these edges will have the same sign as the path they were induced from. Hence, if we compute the TR within the unsigned version of this PG (e.g., by neglecting the signs in the TR step or by setting all signs to “+”) all edges that stem from indirect effects and span one branch of the induced FFLs would still correctly be removed.

Regarding the original negative FFL included in the true graph (Figure 7, left), we cannot be sure which sign it will get in the PG as there is a positive path as well as a negative edge from A to E and, hence, the direction of change in E when perturbing A cannot be predicted uniquely. Only if the overall effect of A on E measured during perturbation of A is negative (i.e., the true edge of A to E is dominating over the positive path from A to E), it may happen that it will be falsely removed during TR if edge signs are neglected. However, when using edge weights for (weighted) TR, it is rather unlikely that the path from A to E fulfills the rule (2) or, for a 2-path used by LTR, rule (3) since the measured overall effect from A to E turned out to be negative, hence, the path seems to have a low potential to transduce an effect from A to E. Thus, it is unlikely that the true edge *A* ⊣ *E* would be falsely removed.

To summarize this aspect, there is a low probability that (weighted) TR removes a true edge within a negative feed-forward loop and neglecting edge signs in TR will therefore have only minor impact on the reconstruction quality. This has important consequences since then the computationally expensive search for the shortest sign-consistent paths (an NP-complete problem) can be safely turned into a simple search for a shortest (unsigned) path connecting a given pair of nodes (a polynomial problem). Thus, when applying TRANSWESD to the 5000-nodes networks, we may then use an exact (*path_exact=1*) instead of an approximate (*path_exact=0*) sub-algorithm for computing shortest signed path. In contrast, *full_check=0* is still required for TRANSWESD in large networks.

3. With LTR and TRANSWESD^s,w,2^ we considered local variants of TR removing an edge only if there is an explaining path of length 2. Whereas TRANSWESD^s,w,2^ performed well for the DREAM challenge but unfavorable for the SysGenSIM data, weighted LTR yielded superior performance in almost all benchmark tests and only (signed or unsigned) TRANSWESD applied to *all* paths could deliver comparable results. We can thus first conclude that using the multiplicative rule (3) is better suited than the max rule (2) when focusing on short paths. However, it remains still paradoxical why TR restricted to paths of length 2 should be sufficient. This can once more be illustrated by Figure 7. If we again assume that the true graph induces a complete PG (i.e., the transitive closure of the true graph as shown on the right-hand side of Figure 7) then we can indeed recognize that there is always a 2-path that can, in principle, explain an edge from an indirect effect (e.g., edge *A* → *E* is explained by the 2-path *A* → *B* → *E*). Hence, in principle, all false positive edges could be identified and removed explaining why LTR exhibits good behavior. However, one has to keep in mind that 2-paths may contain edges that are themselves indirect effects (as *B* → *E* in the example above), hence, the order of edge removal might then become crucial. Here, the strategy to cut lowest-confidence-edges first worked apparently well in the benchmarks.

Again, showing that local TR based on 2-paths does not lead to lower performance has important consequences, as we can then restrict the search on simple triangles whose detection is computationally easier than detecting paths of arbitrary length. In fact, unsigned (signed) LTR required in the average only 8 (9) seconds in networks with 5000 nodes whereas TRANSWESD (in approximation mode!) needed 150 (260) seconds.

4. The SysGenSIM benchmark showed that edge weights really matter to obtain good results with LTR. Since (signed or unsigned) local LTR and unsigned TRANSWESD are computationally feasible in 5000-nodes networks and as they achieved superior results in all benchmarks (outperforming the winning methods of the DREAM4 challenge by far) these techniques appear to be well-suited for the reconstruction of large-scale regulatory networks based on systematic perturbation experiments.

5. Applied to a realistic application scenario with gene expression data from yeast mutants with single knockouts of transcription factors we could demonstrate that our approach delivers a high enrichment of known interactions especially within the top-ranked edge candidates. With this property, our method holds great potential to identify true unknown gene interactions that can subsequently be validated in experiments.

A potential weakness of our PG- and TR-based methods is the requirement to perturb each node in the network at least once. At a genome-scale level, such datasets are currently only available for a small number of organisms. On the other hand, one might focus on smaller sub-networks where all nodes can be perturbed. Furthermore, if *m* nodes out of *n* nodes can be perturbed in a network, we can use the information of the corresponding *m* perturbation experiments to (i) infer the complete sub-network containing only the *m* perturbed nodes and (ii) to infer edges leading from the *m* perturbed nodes to the *n* − *m* unperturbed nodes. In the second of these sub-networks, TR cannot work effectively (no edge will be removed since only single edges and no paths between these nodes exist) meaning that some of the (false positive) edges in the PG reflecting indirect edges cannot be identified as such. However, the provided output might still have its own value and indicate direct or indirect functional relationships. In fact, we employed this approach for the yeast knockout dataset where “only” the TFs were knocked-out.

We also emphasize that perturbation graphs (as a requirement for applying TR) could also be constructed by other approaches than systematic knockouts of all genes. One example are genetical genomics data containing gene expressions measurements from naturally occurring multifactorial perturbations (polymorphisms). As an example for using PG- and TR-based methods based on genetical genomics data see [50].

We noticed that LTR shares some similarities with ARACNE presented in [11]. ARACNE also eliminates an edge in a feed-forward loop consisting of three edges (so-called triplets) if a certain weight condition is fulfilled. However, there are several key differences since ARACNE only operates on undirected and unsigned graphs and uses different weights based on mutual information.

As an important additional results of our study, we have generated new and unprecedented large-scale benchmark datasets that, in contrast to comparable simulations, account for different noise levels. We think that these datasets, which can be downloaded from http://sysgensim.sourceforge.net/datasets.html, are generally useful for unbiased testing of network inference methodologies complementing other available in silico benchmarks.

## Availability of supporting data

The presented inference algorithms and the 5000-gene benchmark (the Pula-Magdeburg single-gene knockout benchmark dataset) can be downloaded from http://sysgensim.sourceforge.net/datasets.html.

## Abbreviations

PG: Perturbation graph; TR: Transitive reduction; LTR: Local transitive reduction; DR-FFL: Down-ranking of feed-forward loop; FFL: Feed-forward loop; TRANSWESD: TRANSitive reduction in WEighted Signed Digraphs; TP: True positive; FP: False positive; FN: False negative.

## Competing interests

The authors declare that they have no competing interests.

## Authors’ contributions

SK and ALF designed the study. AP generated the new datasets. AP and SK implemented the new algorithms. AP, RJF, SH and SK analyzed the data. All authors were involved in the analysis of the benchmark results and in drafting and proofreading the manuscript. All authors read and approved the final manuscript.

## Supplementary Material

Additional file 1 **Supplementary tables and figures.** A collection of additional Tables and Figures is available in Additional_File_1.pdf. Click here for file

Additional file 2 **List of the 300 most confident edges in the reconstructed yeast transcription factor network.** The list of the 300 most confident edges identified with LTR^u,u^ from the yeast knockout dataset is available in Additional_File_2.pdf. Click here for file
