# Supplementary material for: Reconstruction of large-scale regulatory networks based on perturbation graphs and transitive reduction: improved methods and their evaluation
Source: BMC Syst Biol. 2013 Aug 8;7:73. doi: 10.1186/1752-0509-7-73 (PMC4231426; doi:10.1186/1752-0509-7-73)

Additional File 1
Supplementary Tables and Figures

Table T1 - Performance of the inference algorithms on the SysGenSIM networks with connectivity $K \simeq 2$

Each score is the mean of the AUPR computed for the 10 networks with connectivity $K \simeq 2$ simulated according to the same noise configuration. Thresholds used by the inference algorithms are $\beta = 2.0$ and $\gamma = 0.05$ for $\mathrm{PG}^{\mathrm{new}}$, $\alpha = 0.95$ for $\mathrm{TRANSWESD}^{\cdot,\mathrm{w},\infty}$, $\alpha = 1.50$ for $\mathrm{TRANSWESD}^{\cdot,\mathrm{w},2}$ and $\alpha = 0.15$ for $\mathrm{LTR}^{\cdot,\mathrm{w}}$.

	Noise configuration								
Inference algorithm	1 - LL	2 - LM	3 - LH	4 - ML	5 - MM	6 - MH	7 - HL	8 - HM	9 - HH
$\mathrm{PG}^{\mathrm{new}}$	0.6330	0.5841	0.4374	0.5582	0.5473	0.4771	0.4067	0.4154	0.3863
$\mathrm{PG}^{\mathrm{new}}$ + $\mathrm{TRANSWESD}^{\mathrm{u,w},\infty}$	0.6544	0.5886	0.4361	0.5714	0.5580	0.4809	0.4110	0.4194	0.3890
$\mathrm{PG}^{\mathrm{new}}$ + $\mathrm{TRANSWESD}^{\mathrm{s,w},\infty}$	0.6556	0.5894	0.4362	0.5719	0.5586	0.4812	0.4110	0.4193	0.3890
$\mathrm{PG}^{\mathrm{new}}$ + $\mathrm{TRANSWESD}^{\mathrm{u,w},2}$	0.5969	0.4609	0.4055	0.5015	0.4995	0.4366	0.3698	0.3877	0.3629
$\mathrm{PG}^{\mathrm{new}}$ + $\mathrm{TRANSWESD}^{\mathrm{s,w},2}$	0.6011	0.4717	0.4058	0.5036	0.5013	0.4374	0.3704	0.3883	0.3634
$\mathrm{PG}^{\mathrm{new}}$ + $\mathrm{LTR}^{\mathrm{u,u}}$	0.6336	0.5210	0.4188	0.5409	0.5318	0.4594	0.3935	0.4067	0.3770
$\mathrm{PG}^{\mathrm{new}}$ + $\mathrm{LTR}^{\mathrm{s,u}}$	0.6358	0.5270	0.4189	0.5421	0.5328	0.4598	0.3940	0.4071	0.3772
$\mathrm{PG}^{\mathrm{new}}$ + $\mathrm{LTR}^{\mathrm{u,w}}$	0.6644	0.5978	0.4420	0.5709	0.5579	0.4812	0.4106	0.4203	0.3882
$\mathrm{PG}^{\mathrm{new}}$ + $\mathrm{LTR}^{\mathrm{s,w}}$	0.6656	0.5988	0.4420	0.5714	0.5584	0.4814	0.4107	0.4204	0.3883

Table T2 - Performance of the inference algorithms on the SysGenSIM networks with connectivity $K \simeq 2.5$

Each score is the mean of the AUPR computed for the 10 networks with connectivity $K \simeq 2.5$ simulated according to the same noise configuration. Thresholds used by the inference algorithms are $\beta = 2.0$ and $\gamma = 0.05$ for $\mathrm{PG}^{\mathrm{new}}$, $\alpha = 0.95$ for $\mathrm{TRANSWESD}^{\cdot,\mathrm{w},\infty}$, $\alpha = 1.50$ for $\mathrm{TRANSWESD}^{\cdot,\mathrm{w},2}$ and $\alpha = 0.15$ for $\mathrm{LTR}^{\cdot,\mathrm{w}}$.

	Noise configuration								
Inference algorithm	1 - LL	2 - LM	3 - LH	4 - ML	5 - MM	6 - MH	7 - HL	8 - HM	9 - HH
$\mathrm{PG}^{\mathrm{new}}$	0.5432	0.5020	0.3773	0.4745	0.4656	0.4044	0.3309	0.3401	0.3160
$\mathrm{PG}^{\mathrm{new}}$ + $\mathrm{TRANSWESD}^{\mathrm{u,w},\infty}$	0.5547	0.5032	0.3751	0.4821	0.4719	0.4063	0.3338	0.3428	0.3178
$\mathrm{PG}^{\mathrm{new}}$ + $\mathrm{TRANSWESD}^{\mathrm{s,w},\infty}$	0.5559	0.5042	0.3753	0.4829	0.4726	0.4068	0.3340	0.3430	0.3179
$\mathrm{PG}^{\mathrm{new}}$ + $\mathrm{TRANSWESD}^{\mathrm{u,w},2}$	0.4776	0.3659	0.3268	0.3893	0.3938	0.3430	0.2861	0.3045	0.2868
$\mathrm{PG}^{\mathrm{new}}$ + $\mathrm{TRANSWESD}^{\mathrm{s,w},2}$	0.4837	0.3767	0.3275	0.3928	0.3968	0.3445	0.2869	0.3053	0.2875
$\mathrm{PG}^{\mathrm{new}}$ + $\mathrm{LTR}^{\mathrm{u,u}}$	0.5266	0.4305	0.3444	0.4387	0.4341	0.3727	0.3147	0.3285	0.3050
$\mathrm{PG}^{\mathrm{new}}$ + $\mathrm{LTR}^{\mathrm{s,u}}$	0.5292	0.4361	0.3448	0.4406	0.4358	0.3736	0.3152	0.3291	0.3054
$\mathrm{PG}^{\mathrm{new}}$ + $\mathrm{LTR}^{\mathrm{u,w}}$	0.5692	0.5074	0.3736	0.4833	0.4711	0.4045	0.3344	0.3444	0.3190
$\mathrm{PG}^{\mathrm{new}}$ + $\mathrm{LTR}^{\mathrm{s,w}}$	0.5705	0.5083	0.3736	0.4840	0.4717	0.4048	0.3345	0.3444	0.3191

Table T3 - Statistics on edges from inferred SysGenSIM networks (configuration 2 - LM)

The total number of edges and the number of true positives (TPs) and false positives (FPs) for the perturbation graph are shown in the table. The relative reduction of these measures in the graphs obtained after applying the different TR techniques is also displayed. These averaged statistics are computed from the analysis of the graphs obtained after inferring the 30 SysGenSIM networks simulated according to configuration 2 (LM). Thresholds used were $\beta = 2.0$ and $\gamma = 0.05$ for PG^{new}, $\alpha = 0.95$ for $\text{TRANSWESD}^{\cdot,\text{w},\infty}$, $\alpha = 1.50$ for $\text{TRANSWESD}^{\cdot,\text{w},2}$ and $\alpha = 0.15$ for $\text{LTR}^{\cdot,\text{w}}$.

	$K \simeq 1.5$			$K \simeq 2$			$K \simeq 2.5$		
Inference algorithm	Edges	TPs	FPs	Edges	TPs	FPs	Edges	TPs	FPs
PG^{new}	109078	6106	102972	110614	7248	103366	105254	8028	97226
PG^{new} + $\text{TRANSWESD}^{\text{u,w},\infty}$	-1.30%	-1.17%	-1.31%	-2.18%	-1.63%	-2.22%	-4.00%	-2.77%	-4.10%
PG^{new} + $\text{TRANSWESD}^{\text{s,w},\infty}$	-1.25%	-1.08%	-1.26%	-2.06%	-1.39%	-2.11%	-3.85%	-2.41%	-3.96%
PG^{new} + $\text{TRANSWESD}^{\text{u,w},2}$	-20.45%	-24.29%	-20.22%	-24.17%	-32.08%	-23.61%	-26.04%	-38.30%	-25.03%
PG^{new} + $\text{TRANSWESD}^{\text{s,w},2}$	-20.21%	-22.86%	-20.05%	-23.81%	-30.31%	-23.36%	-25.58%	-36.20%	-24.70%
PG^{new} + $\text{LTR}^{\text{u,u}}$	-17.09%	-16.22%	-17.14%	-19.79%	-21.14%	-19.70%	-21.19%	-25.14%	-20.87%
PG^{new} + $\text{LTR}^{\text{s,u}}$	-16.92%	-15.28%	-17.02%	-19.57%	-20.13%	-19.53%	-20.91%	-24.04%	-20.65%
PG^{new} + $\text{LTR}^{\text{u,w}}$	-3.90%	-5.85%	-3.79%	-5.10%	-8.13%	-4.88%	-6.11%	-9.52%	-5.82%
PG^{new} + $\text{LTR}^{\text{s,w}}$	-3.82%	-5.74%	-3.71%	-5.01%	-7.89%	-4.81%	-6.02%	-9.25%	-5.75%

Table T4 - Statistics on edges from inferred SysGenSIM networks (configuration 3 - LH)

The total number of edges and the number of true positives (TPs) and false positives (FPs) for the perturbation graph are shown in the table. The relative reduction of these measures in the graphs obtained after applying the different TR techniques is also displayed. These averaged statistics are computed from the analysis of the graphs obtained after inferring the 30 SysGenSIM networks simulated according to configuration 3 (LH). Thresholds used were $\beta = 2.0$ and $\gamma = 0.05$ for PG^{new}, $\alpha = 0.95$ for $\text{TRANSWESD}^{\cdot,\text{w},\infty}$, $\alpha = 1.50$ for $\text{TRANSWESD}^{\cdot,\text{w},2}$ and $\alpha = 0.15$ for $\text{LTR}^{\cdot,\text{w}}$.

	$K \simeq 1.5$			$K \simeq 2$			$K \simeq 2.5$		
Inference algorithm	Edges	TPs	FPs	Edges	TPs	FPs	Edges	TPs	FPs
PG^{new}	13262	4569	8694	15677	5320	10357	19162	5875	13287
PG^{new} + $\text{TRANSWESD}^{\text{u,w},\infty}$	-2.05%	-1.36%	-2.41%	-2.28%	-1.33%	-2.77%	-2.54%	-1.71%	-2.90%
PG^{new} + $\text{TRANSWESD}^{\text{s,w},\infty}$	-2.03%	-1.34%	-2.40%	-2.25%	-1.30%	-2.74%	-2.44%	-1.61%	-2.80%
PG^{new} + $\text{TRANSWESD}^{\text{u,w},2}$	-12.49%	-12.14%	-12.68%	-17.88%	-18.35%	-17.65%	-22.70%	-24.72%	-21.81%
PG^{new} + $\text{TRANSWESD}^{\text{s,w},2}$	-12.47%	-12.10%	-12.66%	-17.83%	-18.26%	-17.60%	-22.58%	-24.49%	-21.73%
PG^{new} + $\text{LTR}^{\text{u,u}}$	-10.55%	-9.33%	-11.19%	-14.68%	-13.70%	-15.18%	-18.33%	-18.46%	-18.27%
PG^{new} + $\text{LTR}^{\text{s,u}}$	-10.53%	-9.30%	-11.18%	-14.64%	-13.65%	-15.14%	-18.25%	-18.31%	-18.22%
PG^{new} + $\text{LTR}^{\text{u,w}}$	-8.15%	-5.03%	-9.79%	-10.06%	-6.50%	-11.89%	-10.52%	-8.04%	-11.61%
PG^{new} + $\text{LTR}^{\text{s,w}}$	-8.14%	-5.02%	-9.78%	-10.05%	-6.48%	-11.88%	-10.49%	-8.01%	-11.59%

Table T5 - Statistics on edges from inferred SysGenSIM networks (configuration 4 - ML)

The total number of edges and the number of true positives (TPs) and false positives (FPs) for the perturbation graph are shown in the table. The relative reduction of these measures in the graphs obtained after applying the different TR techniques is also displayed. These averaged statistics are computed from the analysis of the graphs obtained after inferring the 30 SysGenSIM networks simulated according to configuration 4 (ML). Thresholds used were $\beta = 2.0$ and $\gamma = 0.05$ for PG^{new}, $\alpha = 0.95$ for $TRANSWESD^{\cdot,w,\infty}$, $\alpha = 1.50$ for $TRANSWESD^{\cdot,w,2}$ and $\alpha = 0.15$ for $LTR^{\cdot,w}$.

	$K \simeq 1.5$			$K \simeq 2$			$K \simeq 2.5$		
Inference algorithm	Edges	TPs	FPs	Edges	TPs	FPs	Edges	TPs	FPs
PG^{new}	13625	5517	8108	20149	6337	13813	28873	6960	21913
PG^{new} + $TRANSWESD^{u,w,\infty}$	-8.98%	-0.31%	-14.88%	-8.86%	-1.03%	-12.45%	-11.50%	-2.13%	-14.47%
PG^{new} + $TRANSWESD^{s,w,\infty}$	-8.77%	-0.23%	-14.59%	-8.55%	-0.86%	-12.09%	-11.12%	-1.79%	-14.08%
PG^{new} + $TRANSWESD^{u,w,2}$	-30.15%	-15.69%	-39.99%	-34.26%	-24.57%	-38.71%	-36.97%	-32.59%	-38.35%
PG^{new} + $TRANSWESD^{s,w,2}$	-30.02%	-15.53%	-39.88%	-33.99%	-24.08%	-38.54%	-36.49%	-31.64%	-38.03%
PG^{new} + $LTR^{u,u}$	-24.23%	-9.32%	-34.38%	-26.60%	-13.97%	-32.40%	-28.55%	-19.11%	-31.54%
PG^{new} + $LTR^{s,u}$	-24.15%	-9.23%	-34.31%	-26.42%	-13.67%	-32.27%	-28.25%	-18.55%	-31.34%
PG^{new} + $LTR^{u,w}$	-21.28%	-4.94%	-32.40%	-21.01%	-6.30%	-27.76%	-19.79%	-7.35%	-23.74%
PG^{new} + $LTR^{s,w}$	-21.24%	-4.90%	-32.36%	-20.93%	-6.19%	-27.70%	-19.67%	-7.11%	-23.66%

Table T6 - Statistics on edges from inferred SysGenSIM networks (configuration 5 - MM)

The total number of edges and the number of true positives (TPs) and false positives (FPs) for the perturbation graph are shown in the table. The relative reduction of these measures in the graphs obtained after applying the different TR techniques is also displayed. These averaged statistics are computed from the analysis of the graphs obtained after inferring the 30 SysGenSIM networks simulated according to configuration 5 (MM). Thresholds used were $\beta = 2.0$ and $\gamma = 0.05$ for PG^{new}, $\alpha = 0.95$ for $TRANSWESD^{\cdot,w,\infty}$, $\alpha = 1.50$ for $TRANSWESD^{\cdot,w,2}$ and $\alpha = 0.15$ for $LTR^{\cdot,w}$.

	$K \simeq 1.5$			$K \simeq 2$			$K \simeq 2.5$		
Inference algorithm	Edges	TPs	FPs	Edges	TPs	FPs	Edges	TPs	FPs
PG^{new}	11599	5373	6226	16898	6152	10746	23738	6742	16996
PG^{new} + $TRANSWESD^{u,w,\infty}$	-8.31%	-0.34%	-15.19%	-8.15%	-1.09%	-12.19%	-9.92%	-1.97%	-13.08%
PG^{new} + $TRANSWESD^{s,w,\infty}$	-8.18%	-0.29%	-15.00%	-7.82%	-0.89%	-11.78%	-9.58%	-1.68%	-12.71%
PG^{new} + $TRANSWESD^{u,w,2}$	-29.44%	-14.49%	-42.34%	-34.18%	-22.78%	-40.71%	-37.14%	-29.93%	-40.00%
PG^{new} + $TRANSWESD^{s,w,2}$	-29.33%	-14.35%	-42.27%	-33.93%	-22.32%	-40.58%	-36.70%	-29.09%	-39.71%
PG^{new} + $LTR^{u,u}$	-23.94%	-9.21%	-36.65%	-26.68%	-13.44%	-34.26%	-28.79%	-18.05%	-33.06%
PG^{new} + $LTR^{s,u}$	-23.88%	-9.14%	-36.60%	-26.53%	-13.18%	-34.17%	-28.50%	-17.53%	-32.86%
PG^{new} + $LTR^{u,w}$	-21.10%	-5.20%	-34.83%	-21.11%	-6.36%	-29.55%	-20.16%	-7.61%	-25.13%
PG^{new} + $LTR^{s,w}$	-21.07%	-5.16%	-34.79%	-21.04%	-6.25%	-29.50%	-20.05%	-7.41%	-25.06%

Table T7 - Statistics on edges from inferred SysGenSIM networks (configuration 6 - MH)

The total number of edges and the number of true positives (TPs) and false positives (FPs) for the perturbation graph are shown in the table. The relative reduction of these measures in the graphs obtained after applying the different TR techniques is also displayed. These averaged statistics are computed from the analysis of the graphs obtained after inferring the 30 SysGenSIM networks simulated according to configuration 6 (MH). Thresholds used were $\beta = 2.0$ and $\gamma = 0.05$ for $\mathrm{PG^{new}}$, $\alpha = 0.95$ for $\mathrm{TRANSWESD^{\cdot,w,\infty}}$, $\alpha = 1.50$ for $\mathrm{TRANSWESD^{\cdot,w,2}}$ and $\alpha = 0.15$ for $\mathrm{LTR^{\cdot,w}}$.

	$K \simeq 1.5$			$K \simeq 2$			$K \simeq 2.5$		
Inference algorithm	Edges	TPs	FPs	Edges	TPs	FPs	Edges	TPs	FPs
$\mathrm{PG^{new}}$	9707	4739	4968	14179	5389	8790	20115	5948	14166
$\mathrm{PG^{new}}$ + $\mathrm{TRANSWESD^{u,w,\infty}}$	-5.16%	-0.50%	-9.61%	-4.71%	-0.87%	-7.06%	-6.35%	-1.63%	-8.33%
$\mathrm{PG^{new}}$ + $\mathrm{TRANSWESD^{s,w,\infty}}$	-5.10%	-0.47%	-9.51%	-4.51%	-0.74%	-6.83%	-6.09%	-1.40%	-8.06%
$\mathrm{PG^{new}}$ + $\mathrm{TRANSWESD^{u,w,2}}$	-22.26%	-12.72%	-31.36%	-26.19%	-19.59%	-30.24%	-28.41%	-26.44%	-29.23%
$\mathrm{PG^{new}}$ + $\mathrm{TRANSWESD^{s,w,2}}$	-22.19%	-12.64%	-31.30%	-26.06%	-19.37%	-30.17%	-28.13%	-25.96%	-29.04%
$\mathrm{PG^{new}}$ + $\mathrm{LTR^{u,u}}$	-18.26%	-8.52%	-27.54%	-20.57%	-12.46%	-25.54%	-22.17%	-17.03%	-24.33%
$\mathrm{PG^{new}}$ + $\mathrm{LTR^{s,u}}$	-18.22%	-8.49%	-27.51%	-20.48%	-12.32%	-25.48%	-21.98%	-16.72%	-24.19%
$\mathrm{PG^{new}}$ + $\mathrm{LTR^{u,w}}$	-15.70%	-4.97%	-25.94%	-15.29%	-5.96%	-21.00%	-14.16%	-6.93%	-17.19%
$\mathrm{PG^{new}}$ + $\mathrm{LTR^{s,w}}$	-15.68%	-4.95%	-25.92%	-15.25%	-5.91%	-20.98%	-14.10%	-6.84%	-17.15%

Table T8 - Statistics on edges from inferred SysGenSIM networks (configuration 7 - HL)

The total number of edges and the number of true positives (TPs) and false positives (FPs) for the perturbation graph are shown in the table. The relative reduction of these measures in the graphs obtained after applying the different TR techniques is also displayed. These averaged statistics are computed from the analysis of the graphs obtained after inferring the 30 SysGenSIM networks simulated according to configuration 7 (HL). Thresholds used were $\beta = 2.0$ and $\gamma = 0.05$ for $\mathrm{PG^{new}}$, $\alpha = 0.95$ for $\mathrm{TRANSWESD^{\cdot,w,\infty}}$, $\alpha = 1.50$ for $\mathrm{TRANSWESD^{\cdot,w,2}}$ and $\alpha = 0.15$ for $\mathrm{LTR^{\cdot,w}}$.

	$K \simeq 1.5$			$K \simeq 2$			$K \simeq 2.5$		
Inference algorithm	Edges	TPs	FPs	Edges	TPs	FPs	Edges	TPs	FPs
$\mathrm{PG^{new}}$	11183	3618	7566	19022	4067	14956	27518	4353	23165
$\mathrm{PG^{new}}$ + $\mathrm{TRANSWESD^{u,w,\infty}}$	-3.07%	-0.12%	-4.49%	-4.00%	-0.33%	-4.99%	-4.84%	-0.71%	-5.62%
$\mathrm{PG^{new}}$ + $\mathrm{TRANSWESD^{s,w,\infty}}$	-3.00%	-0.11%	-4.39%	-3.84%	-0.30%	-4.80%	-4.66%	-0.59%	-5.42%
$\mathrm{PG^{new}}$ + $\mathrm{TRANSWESD^{u,w,2}}$	-14.96%	-9.64%	-17.50%	-18.67%	-17.18%	-19.07%	-19.05%	-21.96%	-18.50%
$\mathrm{PG^{new}}$ + $\mathrm{TRANSWESD^{s,w,2}}$	-14.84%	-9.59%	-17.36%	-18.41%	-16.92%	-18.81%	-18.65%	-21.56%	-18.11%
$\mathrm{PG^{new}}$ + $\mathrm{LTR^{u,u}}$	-11.64%	-5.26%	-14.69%	-14.19%	-8.85%	-15.65%	-14.44%	-11.07%	-15.08%
$\mathrm{PG^{new}}$ + $\mathrm{LTR^{s,u}}$	-11.53%	-5.21%	-14.56%	-13.98%	-8.64%	-15.44%	-14.14%	-10.78%	-14.77%
$\mathrm{PG^{new}}$ + $\mathrm{LTR^{u,w}}$	-9.62%	-2.05%	-13.25%	-9.92%	-2.83%	-11.85%	-9.12%	-3.10%	-10.25%
$\mathrm{PG^{new}}$ + $\mathrm{LTR^{s,w}}$	-9.59%	-2.03%	-13.21%	-9.86%	-2.79%	-11.78%	-9.05%	-3.05%	-10.18%

Table T9 - Statistics on edges from inferred SysGenSIM networks (configuration 8 - HM)

The total number of edges and the number of true positives (TPs) and false positives (FPs) for the perturbation graph are shown in the table. The relative reduction of these measures in the graphs obtained after applying the different TR techniques is also displayed. These averaged statistics are computed from the analysis of the graphs obtained after inferring the 30 SysGenSIM networks simulated according to configuration 8 (HM). Thresholds used were $\beta = 2.0$ and $\gamma = 0.05$ for PG^{new}, $\alpha = 0.95$ for $TRANSWESD^{\cdot,w,\infty}$, $\alpha = 1.50$ for $TRANSWESD^{\cdot,w,2}$ and $\alpha = 0.15$ for $LTR^{\cdot,w}$.

	$K \simeq 1.5$			$K \simeq 2$			$K \simeq 2.5$		
Inference algorithm	Edges	TPs	FPs	Edges	TPs	FPs	Edges	TPs	FPs
PG^{new}	8247	3526	4721	13383	3966	9417	19019	4264	14755
PG^{new} + $TRANSWESD^{u,w,\infty}$	-3.60%	-0.11%	-6.21%	-3.72%	-0.35%	-5.14%	-4.48%	-0.61%	-5.60%
PG^{new} + $TRANSWESD^{s,w,\infty}$	-3.55%	-0.10%	-6.13%	-3.53%	-0.32%	-4.88%	-4.23%	-0.47%	-5.31%
PG^{new} + $TRANSWESD^{u,w,2}$	-17.68%	-8.56%	-24.49%	-22.21%	-15.55%	-25.01%	-22.93%	-20.02%	-23.77%
PG^{new} + $TRANSWESD^{s,w,2}$	-17.64%	-8.52%	-24.46%	-22.05%	-15.32%	-24.88%	-22.68%	-19.65%	-23.56%
PG^{new} + $LTR^{u,u}$	-13.70%	-4.78%	-20.36%	-16.73%	-8.07%	-20.38%	-17.08%	-10.03%	-19.12%
PG^{new} + $LTR^{s,u}$	-13.67%	-4.74%	-20.34%	-16.62%	-7.93%	-20.28%	-16.90%	-9.78%	-18.96%
PG^{new} + $LTR^{u,w}$	-11.55%	-1.96%	-18.72%	-11.85%	-2.76%	-15.68%	-11.22%	-3.09%	-13.57%
PG^{new} + $LTR^{s,w}$	-11.54%	-1.95%	-18.71%	-11.82%	-2.72%	-15.65%	-11.18%	-3.04%	-13.53%

Table T10 - Statistics on edges from inferred SysGenSIM networks (configuration 9 - HH)

The total number of edges and the number of true positives (TPs) and false positives (FPs) for the perturbation graph are shown in the table. The relative reduction of these measures in the graphs obtained after applying the different TR techniques is also displayed. These averaged statistics are computed from the analysis of the graphs obtained after inferring the 30 SysGenSIM networks simulated according to configuration 9 (HH). Thresholds used were $\beta = 2.0$ and $\gamma = 0.05$ for PG^{new}, $\alpha = 0.95$ for $TRANSWESD^{\cdot,w,\infty}$, $\alpha = 1.50$ for $TRANSWESD^{\cdot,w,2}$ and $\alpha = 0.15$ for $LTR^{\cdot,w}$.

	$K \simeq 1.5$			$K \simeq 2$			$K \simeq 2.5$		
Inference algorithm	Edges	TPs	FPs	Edges	TPs	FPs	Edges	TPs	FPs
PG^{new}	7396	3181	4215	12084	3625	8459	17230	3932	13298
PG^{new} + $TRANSWESD^{u,w,\infty}$	-2.70%	-0.09%	-4.67%	-3.36%	-0.29%	-4.67%	-3.48%	-0.54%	-4.35%
PG^{new} + $TRANSWESD^{s,w,\infty}$	-2.64%	-0.08%	-4.58%	-3.20%	-0.28%	-4.46%	-3.26%	-0.47%	-4.08%
PG^{new} + $TRANSWESD^{u,w,2}$	-13.78%	-7.44%	-18.56%	-18.19%	-13.64%	-20.14%	-18.68%	-17.38%	-19.07%
PG^{new} + $TRANSWESD^{s,w,2}$	-13.74%	-7.39%	-18.54%	-18.05%	-13.45%	-20.03%	-18.43%	-17.02%	-18.85%
PG^{new} + $LTR^{u,u}$	-10.91%	-4.29%	-15.91%	-14.01%	-7.72%	-16.71%	-14.08%	-9.26%	-15.51%
PG^{new} + $LTR^{s,u}$	-10.88%	-4.23%	-15.89%	-13.93%	-7.62%	-16.63%	-13.90%	-9.03%	-15.33%
PG^{new} + $LTR^{u,w}$	-9.01%	-1.89%	-14.38%	-9.72%	-3.01%	-12.59%	-8.85%	-2.73%	-10.66%
PG^{new} + $LTR^{s,w}$	-9.00%	-1.89%	-14.37%	-9.68%	-2.97%	-12.56%	-8.82%	-2.70%	-10.62%

Figure F1 - Performance and robustness of the perturbation graph methodology PG^{new} applied to the 9 noise configurations of network 11 in the SysGenSIM dataset

The AUPR scores of PG^{new} are fairly robust for a large range of meaningful parameter values β and γ. The picture shows the performance of the inference of Network 11 (containing about 10000 edges, i.e. $K \simeq 2$) with respect to the 9 different noise conditions.

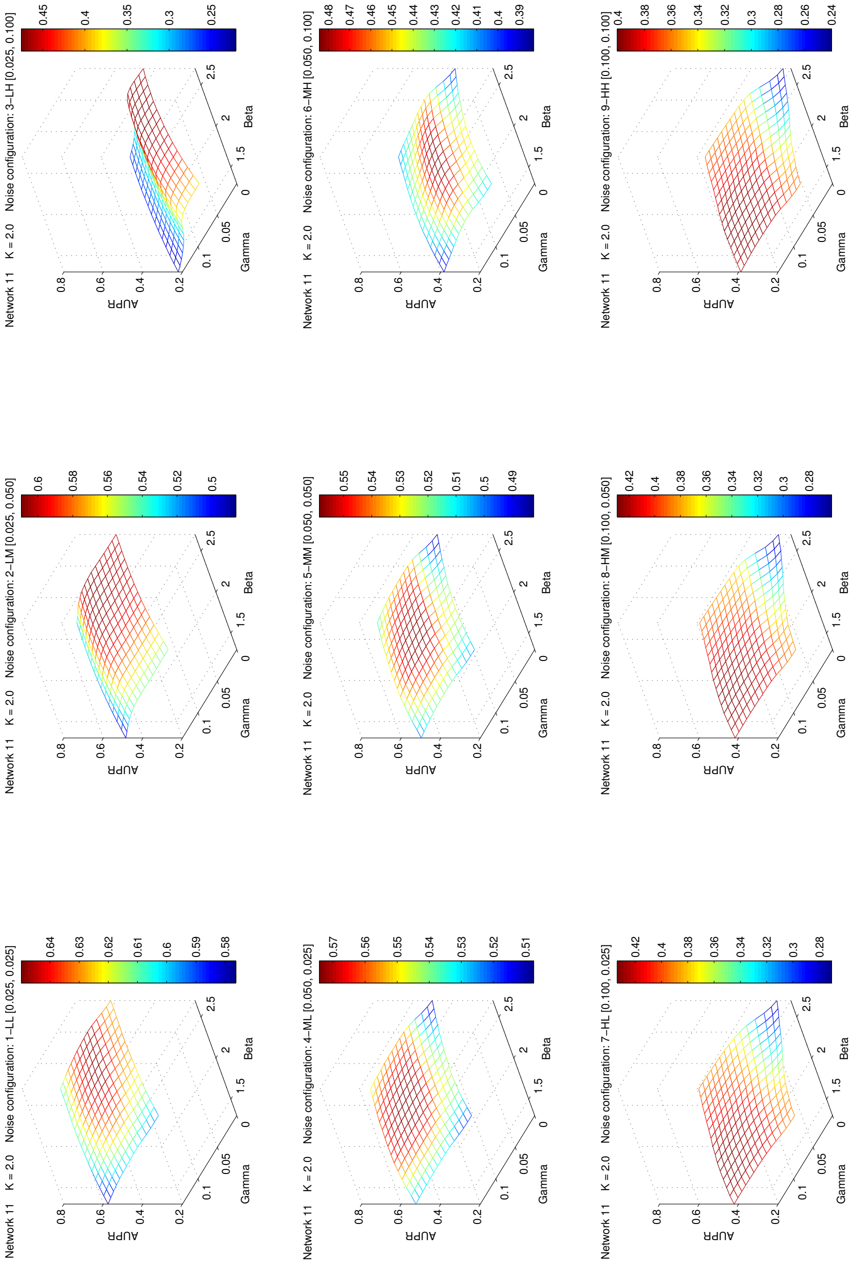

Figure F2 - Performance and robustness of the perturbation graph methodology PG^{new} applied to the 9 noise configurations of network 21 in the SysGenSIM dataset

The AUPR scores of PG^{new} are fairly robust for a large range of meaningful parameter values β and γ. The picture shows the performance of the inference of Network 21 (containing about 12500 edges, i.e. $K \simeq 2.5$) with respect to the 9 different noise conditions.

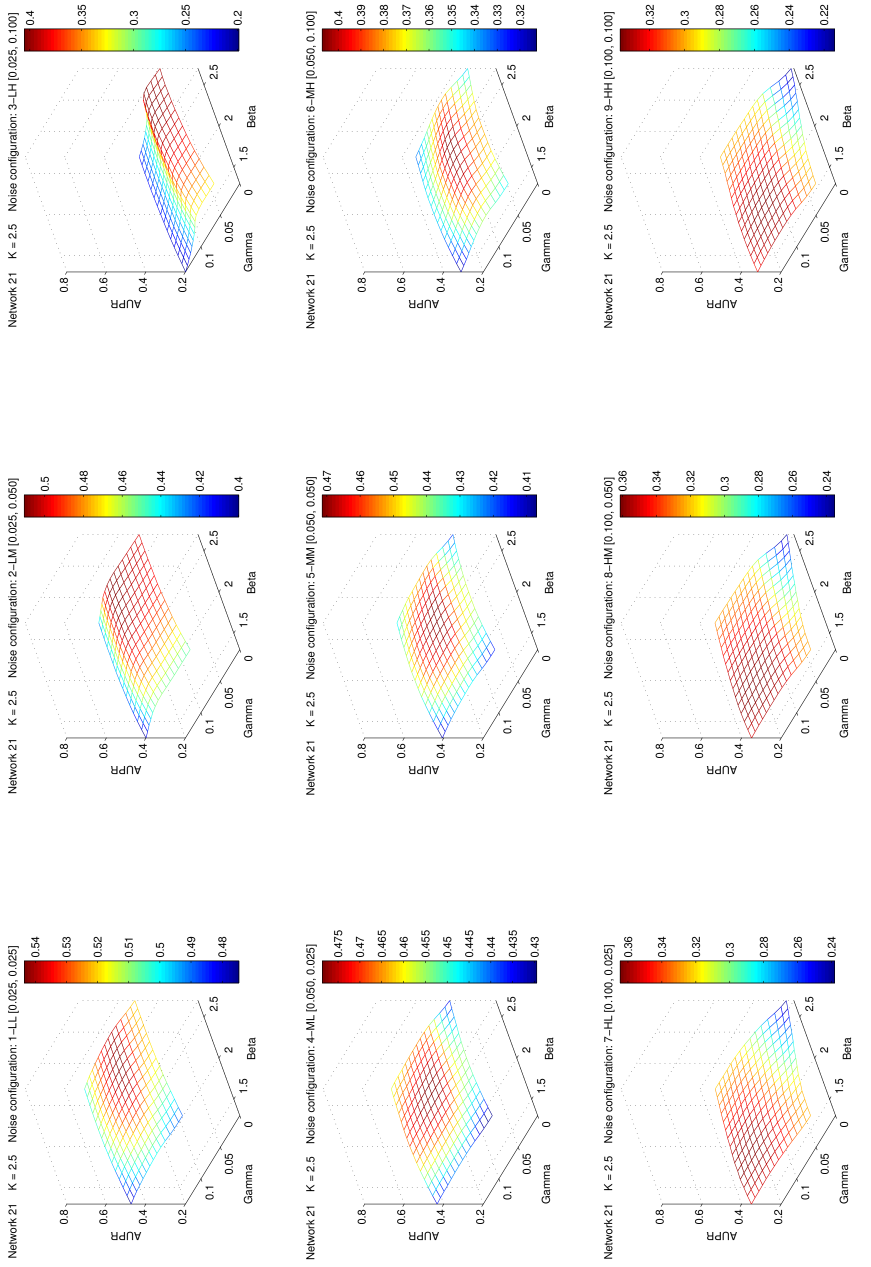

Figure F3 - Performance of the new TRANSWESD and LTR variants on the SysGenSIM dataset

Parameters used to obtain the perturbation graph were $\beta = 2.0$ and $\gamma = 0.05$, while $\alpha = 0.95$ and $\alpha = 0.15$ were selected for the TRANSWESD and LTR variants, respectively. AUPR scores are averaged across the 10 networks generated with the same K and simulated with the same noise configuration. The efficacy of transitive reduction changes with the connectivity of the network and with the noise levels.

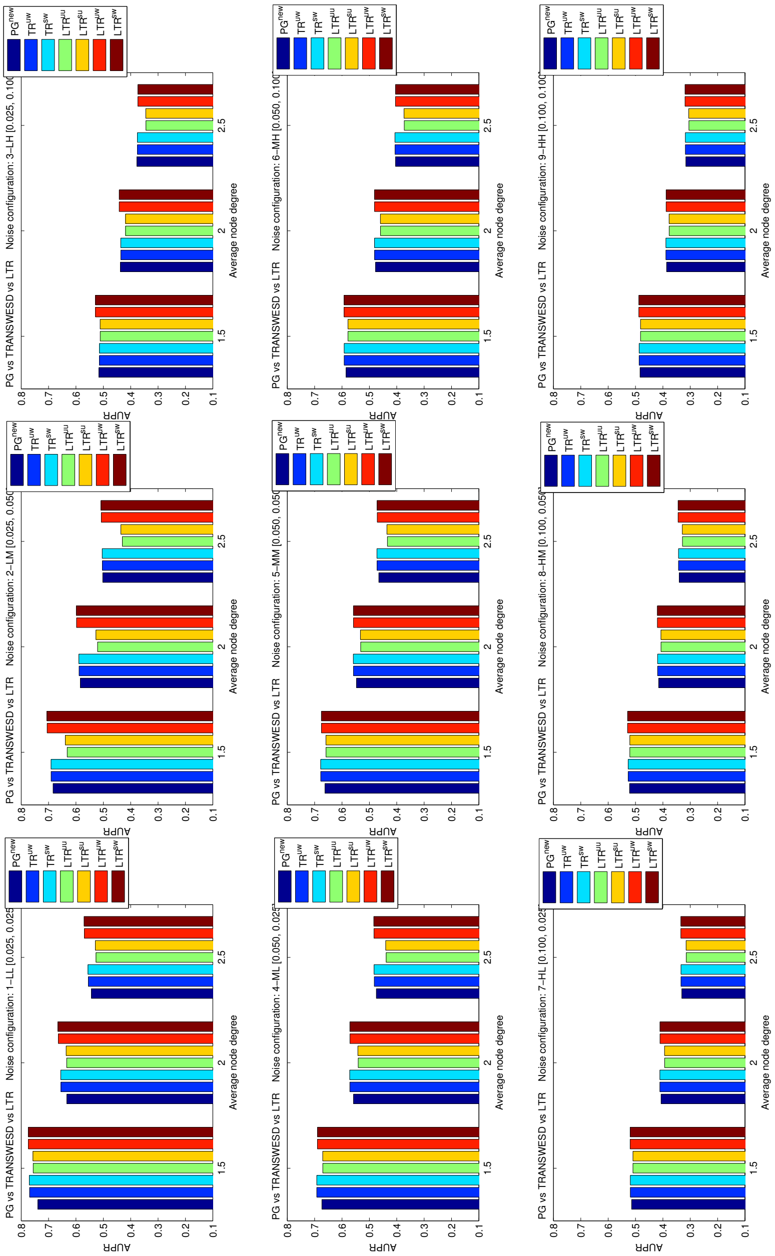

Supplement: Additional file 1 — Supplementary tables and figures. A collection of additional Tables and Figures is available in Additional_File_1.pdf. [file 1752-0509-7-73-S1.pdf]
