# Supplementary material for: Reconstruction of large-scale regulatory networks based on perturbation graphs and transitive reduction: improved methods and their evaluation
Source: BMC Syst Biol. 2013 Aug 8;7:73. doi: 10.1186/1752-0509-7-73 (PMC4231426; doi:10.1186/1752-0509-7-73)
Supplement: Additional file 2 — List of the 300 most confident edges in the reconstructed yeast transcription factor network. The list of the 300 most confident edges identified with LTRu,u from the yeast knockout dataset is available in Additional_File_2.pdf. [file 1752-0509-7-73-S2.pdf]

Additional File 2

Table T4 - List of predicted gene interactions in *S. cerevisiae* obtained from a transcription factor knockout dataset

The table shows a sorted list of the 300 most confident edges obtained by applying PG^{new} and $LTR^{u,u}$ to the dataset published by Reimand et al. in `http://www.ebi.ac.uk/arrayexpress/experiments/E-MTAB-109`. Each predicted edge is compared against the four silver standards $\mathbf{SS}_1$-$\mathbf{SS}_4$ (see main text).

The values in the silver standard columns are:

1: if the predicted edge is contained in the silver standard;

0: if the predicted edge is not contained in the silver standard;

x: if the predicted edge contains a node not contained in the silver standard.

Ranking	Transcription factor	Target gene	$\mathbf{SS}_1$	$\mathbf{SS}_2$	$\mathbf{SS}_3$	$\mathbf{SS}_4$
1	YML051W	YBR018C	1	1	x	0
2	YML051W	YBR020W	1	0	x	1
3	YML051W	YBR019C	1	0	x	1
4	YLR176C	YML058W.A	0	0	1	0
5	YHR006W	YHR049C.A	0	0	x	x
6	YLR176C	YMR279C	1	1	1	0
7	YLR176C	YIL066C	1	1	1	1
8	YBR150C	YIR027C	x	x	x	x
9	YML051W	YLR081W	1	1	x	0
10	YIL038C	YDR198C	x	x	x	x
11	YMR070W	YJR150C	0	0	0	0
12	YBR033W	YNR050C	0	0	x	x
13	YHL020C	YJL153C	0	0	x	0
14	YOR191W	YDL179W	x	x	x	x
15	YLR278C	YKL134C	x	x	x	x
16	YPR065W	YDR044W	1	0	1	0
17	YHL027W	YLL053C	0	0	0	0
18	YDR310C	YNL318C	1	1	1	1
19	YHL027W	YPL277C	0	0	0	0
20	YOR038C	YPR089W	0	0	x	x
21	YBL103C	YCR097W	1	0	1	0
22	YHL027W	YPL278C	0	0	0	0
23	YGL073W	YPL111W	0	0	0	0
24	YOR162C	YCL002C	0	0	x	0
25	YDR310C	YCL048W	1	1	1	1
26	YPL254W	YGL091C	x	x	x	x
27	YDR310C	YNL319W	0	0	x	x
28	YHL027W	YOR389W	0	0	0	0
29	YLR176C	YGR180C	1	0	1	1
30	YDR207C	YOL131W	1	1	x	1
31	YOL067C	YGR077C	0	0	0	x
32	YOL067C	YFR021W	0	0	x	x
33	YDR310C	YFR032C	1	1	1	1
34	YJR094C	YPR164W	0	0	0	0
35	YOR140W	YKR102W	x	x	0	x
36	YGL237C	YEL013W	0	0	0	0
37	YDR310C	YAL018C	1	1	0	0
38	YFL052W	YAR030C	0	0	x	x
39	YKL112W	YBR238C	0	0	0	0
40	YLR176C	YJL026W	0	0	1	0
41	YCR084C	YMR325W	x	x	x	x
42	YBR033W	YNL032W	0	0	x	x
43	YDL020C	YGR252W	0	0	0	0
44	YDL020C	YDR172W	0	0	x	0
45	YDR207C	YAL054C	1	0	1	1
46	YGR056W	YLL054C	x	x	x	x
47	YCR084C	YAL068C	x	x	x	x
48	YCR084C	YHL046C	x	x	x	x
49	YCR084C	YGR294W	x	x	x	x
50	YCR084C	YDR542W	x	x	x	x
51	YBR033W	YBR291C	0	0	x	x
52	YOR162C	YBL037W	0	0	x	0
53	YCR084C	YLR461W	x	x	x	x
54	YCR084C	YCR104W	x	x	x	x
55	YCR084C	YNR076W	x	x	x	x
56	YDR310C	YGL138C	1	1	1	1
57	YCR084C	YIL176C	x	x	x	x
58	YCR084C	YFL020C	x	x	x	x
59	YJL168C	YNR062C	x	x	x	x
60	YCR084C	YAR020C	x	x	x	x

Ranking	Transcription factor	Target gene	$\mathbf{SS}_1$	$\mathbf{SS}_2$	$\mathbf{SS}_3$	$\mathbf{SS}_4$
61	YCR084C	YOL161C	x	x	x	x
62	YDR310C	YJL043W	1	0	x	1
63	YBR049C	YER187W.A	0	0	x	x
64	YDR207C	YIL072W	1	1	1	0
65	YLR176C	YPR015C	0	0	x	0
66	YJL176C	YMR011W	x	x	0	x
67	YDR310C	YCL026C.A	0	0	0	0
68	YJL176C	YDR043C	x	x	0	x
69	YCR084C	YAL064W.B	x	x	x	x
70	YBR049C	YLR113W	1	0	0	0
71	YCR084C	YIL172C	x	x	x	x
72	YGL073W	YIR031C	0	0	0	0
73	YIR033W	YMR169C	x	x	x	x
74	YBR033W	YIR034C	0	0	x	x
75	YDR310C	YOL132W	1	1	0	0
76	YCR084C	YAL065C	x	x	x	x
77	YDR207C	YNL012W	1	1	0	0
78	YHL020C	YER026C	0	0	x	0
79	YPL075W	YIL082W	0	0	0	x
80	YDR310C	YOL091W	1	1	0	0
81	YMR043W	YER037W	0	0	x	0
82	YIL084C	YLR303W	x	x	x	x
83	YDR310C	YBR180W	1	1	1	1
84	YCR084C	YKL224C	x	x	x	x
85	YCR084C	YJL223C	x	x	x	x
86	YBR033W	YIL094C	0	0	x	x
87	YCR084C	YOL157C	x	x	x	x
88	YCR084C	YJR158W	x	x	x	x
89	YMR043W	YKR075C	0	0	0	0
90	YBR049C	YNL143C	0	0	x	x
91	YHL020C	YDR502C	0	0	x	0
92	YOL148C	YOR113W	x	x	x	x
93	YJL176C	YGR161C	x	x	0	x
94	YDR310C	YBL098W	0	0	0	0
95	YJL103C	YGR222W	x	x	x	x
96	YDR310C	YLR307W	0	0	x	0
97	YMR043W	YBL069W	0	0	x	0
98	YGL073W	YKL163W	0	0	0	0
99	YCR084C	YIR041W	x	x	x	x
100	YNL216W	YBR033W	0	0	0	0
101	YML099C	YOL140W	1	1	1	1
102	YIR033W	YML130C	x	x	x	x
103	YJL176C	YKL096W	x	x	0	x
104	YDR207C	YIL031W	1	1	0	0
105	YDR207C	YCR010C	1	0	x	0
106	YDL042C	YNL337W	x	x	x	x
107	YDR520C	YBL042C	0	0	x	0
108	YJL127C	YNL332W	x	x	x	x
109	YDR191W	YIR038C	x	x	x	x
110	YDR310C	YPL033C	1	0	x	0
111	YNL236W	YMR031W.A	x	x	x	x
112	YMR043W	YER130C	0	0	0	0
113	YHL020C	YDR497C	1	1	x	0
114	YDR310C	YOR365C	1	1	1	1
115	YNL167C	YBL071C	0	0	0	x
116	YML099C	YJL088W	1	0	1	1
117	YJL176C	YBR230C	x	x	0	x
118	YBR297W	YGR269W	x	x	x	x
119	YDR207C	YLL047W	0	0	x	x
120	YCR084C	YBR301W	x	x	x	x

Ranking	Transcription factor	Target gene	$\mathbf{SS}_1$	$\mathbf{SS}_2$	$\mathbf{SS}_3$	$\mathbf{SS}_4$
121	YDR310C	YOR339C	1	1	x	0
122	YML099C	YOL058W	1	0	1	1
123	YDR310C	YBR045C	1	1	x	0
124	YDR207C	YPL018W	1	0	0	0
125	YIL130W	YPR109W	x	x	x	x
126	YDR310C	YGR259C	0	0	0	x
127	YDR310C	YGR260W	1	1	1	1
128	YPL075W	YHR046C	0	0	0	0
129	YCR084C	YFL053W	x	x	x	x
130	YIL036W	YBR116C	0	0	x	x
131	YDR310C	YOL047C	1	1	x	0
132	YMR042W	YOL058W	0	0	1	0
133	YMR042W	YOL140W	1	1	1	1
134	YCR084C	YHR211W	x	x	x	x
135	YCR084C	YBR300C	x	x	x	x
136	YDR207C	YGL033W	1	0	0	0
137	YOL004W	YEL020C	x	x	0	x
138	YHL027W	YBR182C	0	0	0	0
139	YCR084C	YIL099W	x	x	x	x
140	YDR207C	YMR306C.A	0	0	0	x
141	YJL089W	YKR071C	0	0	0	0
142	YIR033W	YMR170C	x	x	x	x
143	YIL131C	YAL034W.A	1	1	x	0
144	YDR207C	YMR101C	1	1	x	1
145	YJL127C	YMR096W	x	x	x	x
146	YLR176C	YPL189W	0	0	x	0
147	YLR403W	YPL095C	0	0	0	0
148	YJL127C	YJR156C	x	x	x	x
149	YDR310C	YOR214C	0	0	x	0
150	YNL216W	YOR292C	0	0	1	0
151	YMR043W	YBR054W	1	0	0	0
152	YDR310C	YKR015C	1	1	x	0
153	YKL112W	YDL039C	0	0	0	0
154	YMR043W	YJL016W	0	0	x	0
155	YGL073W	YNL160W	0	0	x	0
156	YMR043W	YER053C	0	0	x	0
157	YIR033W	YMR040W	x	x	x	x
158	YMR043W	YKR058W	0	0	x	0
159	YIR033W	YOL031C	x	x	x	x
160	YOL004W	YER119C	x	x	x	x
161	YIR033W	YOR289W	x	x	x	x
162	YMR043W	YPL188W	0	0	0	0
163	YDR463W	YFR030W	0	0	0	0
164	YBL103C	YLR089C	1	0	0	0
165	YHL020C	YJR073C	0	0	x	0
166	YDR310C	YPR054W	1	1	1	0
167	YLR403W	YBR157C	0	0	0	0
168	YCR084C	YLL055W	x	x	x	x
169	YNL167C	YOL150C	0	0	x	x
170	YOL108C	YNL105W	0	0	x	x
171	YDR310C	YJL038C	1	0	1	0
172	YOR290C	YOR179C	x	x	x	x
173	YPL075W	YNR056C	0	0	x	0
174	YJL168C	YNR057C	x	x	x	x
175	YPL254W	YMR031W.A	x	x	x	x
176	YCR084C	YOR009W	x	x	x	x
177	YHL020C	YNL170W	0	0	x	x
178	YKL043W	YPL120W	0	0	x	0
179	YIR033W	YMR105C	x	x	x	x
180	YHL020C	YLR133W	0	0	x	0

Ranking	Transcription factor	Target gene	$\mathbf{SS}_1$	$\mathbf{SS}_2$	$\mathbf{SS}_3$	$\mathbf{SS}_4$
181	YJL176C	YCR020C	x	x	x	x
182	YLR403W	YHL028W	0	0	0	0
183	YGL073W	YHL043W	0	0	x	0
184	YIR033W	YHR087W	x	x	x	x
185	YJL176C	YDR137W	x	x	x	x
186	YIR033W	YDL124W	x	x	x	x
187	YDR207C	YHR202W	1	1	0	0
188	YMR043W	YJR096W	0	0	x	0
189	YDR310C	YJR025C	1	1	1	1
190	YDR207C	YLL030C	1	1	x	x
191	YPL075W	YGR109W.A	0	0	0	0
192	YPL075W	YLR432W	0	0	x	0
193	YCR084C	YDR402C	x	x	x	x
194	YNL236W	YOR218C	x	x	x	x
195	YPL075W	YHR216W	0	0	x	0
196	YHL020C	YLR132C	0	0	x	0
197	YLR228C	YLR165C	x	x	x	x
198	YDR310C	YNL204C	1	1	0	0
199	YJL176C	YCL040W	x	x	0	x
200	YCR084C	YGL158W	x	x	x	x
201	YNL314W	YHR066W	0	0	x	0
202	YKL112W	YLR162W	0	0	x	0
203	YDR207C	YHR014W	1	1	1	0
204	YMR016C	YKL043W	1	0	0	1
205	YIL036W	YDR453C	0	0	x	0
206	YBR289W	YDR416W	x	x	x	x
207	YGL073W	YGR121C	0	0	x	0
208	YDR310C	YBR148W	1	1	1	1
209	YNL216W	YHR156C	0	0	0	0
210	YMR043W	YDR222W	0	0	x	0
211	YHL027W	YLL052C	0	0	0	0
212	YOL148C	YHR110W	x	x	x	x
213	YLR039C	YGL229C	x	x	x	x
214	YKL032C	YGL250W	0	0	x	0
215	YLR403W	YOR338W	0	0	0	0
216	YCR081W	YIL009W	x	x	x	x
217	YOR162C	YNL195C	0	0	x	0
218	YDR310C	YER085C	1	1	0	0
219	YDR310C	YOR364W	0	0	x	x
220	YNL314W	YOL138C	0	0	0	0
221	YPL075W	YIL083C	0	0	0	0
222	YDR310C	YMR244W	0	0	0	0
223	YOL004W	YEL012W	x	x	0	x
224	YCR084C	YGR293C	x	x	x	x
225	YPL075W	YAR075W	0	0	x	0
226	YMR043W	YER150W	1	0	x	0
227	YNL167C	YOL151W	1	1	0	0
228	YOL051W	YPL181W	x	x	x	x
229	YJL127C	YNR064C	x	x	x	x
230	YDL020C	YJL163C	0	0	x	0
231	YHL020C	YGL077C	0	0	x	0
232	YIL036W	YDR034W.B	0	0	x	0
233	YDR310C	YOR190W	1	1	x	0
234	YIL036W	YAL061W	0	0	x	0
235	YLR039C	YOR040W	x	x	x	x
236	YMR273C	YGL100W	x	x	x	x
237	YNL236W	YHL043W	x	x	x	x
238	YGR288W	YLR312W.A	x	x	x	x
239	YPR065W	YJR047C	1	1	1	0
240	YJL176C	YCR047C	x	x	0	x

Ranking	Transcription factor	Target gene	$\mathbf{SS}_1$	$\mathbf{SS}_2$	$\mathbf{SS}_3$	$\mathbf{SS}_4$
241	YML099C	YER069W	1	1	1	1
242	YLR442C	YDR449C	x	x	x	x
243	YMR042W	YER069W	1	1	1	1
244	YDR310C	YKR034W	0	0	0	0
245	YIR033W	YOL084W	x	x	x	x
246	YJL176C	YOR374W	x	x	x	x
247	YOL068C	YJR025C	x	x	x	x
248	YDR310C	YFR023W	1	1	1	1
249	YJL176C	YCR004C	x	x	x	x
250	YJL176C	YER062C	x	x	0	x
251	YDR207C	YPR007C	1	1	x	0
252	YMR053C	YDR189W	0	0	x	0
253	YLR014C	YKL170W	0	0	x	x
254	YDR207C	YBR302C	0	0	x	0
255	YOL004W	YER044C	x	x	0	x
256	YPL129W	YJR103W	x	x	x	x
257	YOL148C	YJR162C	x	x	x	x
258	YER040W	YDR496C	0	0	x	0
259	YOR191W	YKL100C	x	x	x	x
260	YOL089C	YLL028W	0	0	0	x
261	YPL075W	YFL029C	0	0	x	0
262	YIR033W	YHR104W	x	x	x	x
263	YMR043W	YER067W	0	0	x	0
264	YCR084C	YCL069W	x	x	x	x
265	YDR207C	YNR057C	0	0	0	0
266	YDR207C	YHL048W	0	0	0	0
267	YLR403W	YPL159C	0	0	0	0
268	YOL068C	YGL230C	x	x	x	x
269	YJL127C	YNL335W	x	x	x	x
270	YHR006W	YER008C	0	0	x	x
271	YCR084C	YML131W	x	x	x	x
272	YPR022C	YBR281C	x	x	x	x
273	YOR140W	YBR260C	x	x	x	x
274	YCR084C	YJR159W	x	x	x	x
275	YMR280C	YER066W	x	x	0	x
276	YMR037C	YLR323C	0	0	x	0
277	YKR036C	YMR158W	x	x	x	x
278	YOR290C	YLR092W	x	x	x	x
279	YDR207C	YHL024W	1	0	0	0
280	YOR290C	YOR119C	x	x	x	x
281	YDR310C	YGL015C	1	1	0	0
282	YCR084C	YAR062W	x	x	x	x
283	YLR418C	YAL062W	x	x	x	x
284	YLR418C	YJL171C	x	x	x	x
285	YBR289W	YIR017C	x	x	x	x
286	YMR042W	YJL088W	1	0	1	1
287	YJL168C	YLR087C	x	x	x	x
288	YPL075W	YDL066W	0	0	0	0
289	YOL068C	YLR307W	x	x	x	x
290	YGL181W	YNL021W	0	0	x	0
291	YNL216W	YOR394W	0	0	0	0
292	YNL314W	YMR039C	0	0	0	0
293	YMR273C	YEL030W	x	x	x	x
294	YLR182W	YIL120W	0	0	x	0
295	YGL073W	YBR067C	0	0	0	0
296	YDR310C	YER106W	1	1	x	0
297	YHL020C	YNL256W	0	0	x	0
298	YJL127C	YLR217W	x	x	x	x
299	YGL073W	YJL079C	0	0	0	0
300	YBL008W	YPR109W	0	0	x	x
